# Role of Nitrate-Driven Radical Formation in Microorganism Inactivation under 222 nm UV Irradiation

**DOI:** 10.1021/acsestwater.5c01533

**Published:** 2026-05-28

**Authors:** Dana Pousty, Emma M. Payne, Karl G. Linden

**Affiliations:** Department of Civil, Environmental, and Architectural Engineering, 1877 University of Colorado Boulder, 4001 Discovery Dr., Boulder, Colorado 80303, United States

**Keywords:** Far-UV, KrCl* excimer, nitrate photolysis, bacteria and virus disinfection, hydroxyl radical, reactive nitrogen species

## Abstract

Far-UVC at 222 nm is a promising alternative to conventional UV at 254 nm, offering potent antimicrobial efficacy and in situ oxidation via radical generation from water constituents such as nitrate. However, the role of nitrate-derived reactive species in microbial inactivation remains unclear. This study quantitatively evaluates the impact of nitrate-driven radical production by Far-UVC on microbial disinfection using krypton chloride (KrCl*) excimer lamps. MS2 and T1UV bacteriophage and*Pseudomonas aeruginosa* inactivation were evaluated at environmentally relevant nitrate concentrations (0–8 mg N L^–1^). For MS2, 222 nm achieved higher inactivation rates than 254 nm, with 4 mg N L^–1^ nitrate significantly enhancing reduction, attributed to radical production from nitrate photolysis. Quenching with *tert*-butyl alcohol (TBA) confirmed hydroxyl radical (•OH) as the dominant species, while reactive nitrogen species (RNS) contributed minimally. T1UV exhibited high intrinsic sensitivity to 222 nm direct photolysis, and *P. aeruginosa* showed negligible enhancement from radicals, indicating limited oxidative contribution. Apparent biomolecular rate constants, quantified for MS2 and T1UV, were 1.60–5.14 × 10^10^ M^–1^ s^–1^ for •OH and 8.79 × 10^4^–1.46 × 10^5^ M^–1^ s^–1^ for RNS. Coupled with radical kinetic modeling, these findings demonstrate that •OH governs oxidative effects in Far-UVC/nitrate systems for microorganisms, with implications for the treatment of nitrate-containing wastewater and water.

## Graphical abstract

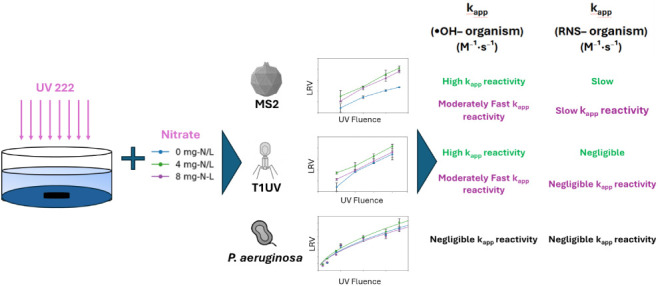

## 1 Introduction

Growing global water scarcity has driven the pursuit of sustainable water reuse practices, reflecting the urgent need for efficient and reliable treatment and disinfection of impaired waters including reclaimed wastewater effluent. Reclaimed water sources often contain human pathogenic viruses and bacteria, posing public health risks if not properly treated. Ultraviolet (UV) light is a commonly used disinfection method with advantages over conventional chemical disinfection (such as chlorination or ozonation), such as being a chemical-free process, not forming chlorinated disinfection byproducts (DBPs), requiring no transportation, storage, or handling of toxic or corrosive chemicals, and not resulting in disinfectant-resistant bacteria.^1–3^

Low-pressure (LP) mercury-vapor UV lamps emitting at 254 nm (UV254) are widely used in water disinfection due to their cost-effectiveness, long lamp life (up to 16,000 h), and relatively high energy efficiency, with 30–35% of electrical input converted into germicidal 254 nm UVC photons.^4^ The LPUV monochromatic emission aligns closely with the DNA absorption peak, making it effective for genetic damage-based inactivation.^5,6^ However, LP lamps lack a broader spectral emission necessary to disrupt viral protein structure or induce oxidative stress and are not highly effective for certain pathogens like adenovirus.^7^ Furthermore, LP lamps contain mercury, presenting environmental and safety concerns, including fragility and mercury toxicity risks,^8^ damage to skin and eyes in cases of accidental exposure, and the time from ignition to full power before usage.^9^

Far-UVC light generated by krypton chloride excimer (KrCl*) lamps which emit primarily at 222 nm (UV222) offers a promising alternative to conventional mercury-based UV systems. Unlike traditional 254 nm lamps, 222 nm KrCl* emission can damage both nucleic acids and protein structures due to its strong absorbance by both nucleic acids and peptides, resulting in enhanced inactivation of viruses, including chlorine-resistant strains.^10^ Recent studies have shown that UV222 can enhance bacteria and virus disinfection compared to UV254 LP lamps^10–13^ and accelerate chemical contaminant degradation.^14–16^ In cases where UV exposure may be a concern, Far-UVC is considered safer for human exposure because it does not penetrate the outer dead layer of the skin or the tear layer of the eyes, making it suitable for occupied spaces.^17^ However, disadvantages include higher operational costs, lower energy efficiency and limited photon penetration compared to UV254. In the presence of nitrate in water, there is also potential UV222 driven formation of nitrite and toxic nitration byproducts.^18^

Nitrate exhibits a high molar absorptivity at 222 nm (ε = 2747 M^–1^ cm^–1^),^15^ making KrCl* excimer systems an effective promoter for advanced oxidation processes (AOPs). Nitrate photolysis produces reactive oxygen species (ROS) such as hydroxyl radicals (•OH) and reactive nitrogen species (RNS) like NO_2_• and peroxynitrite (ONOO^–^) with high quantum yields (Φ­(•OH) = 0.113, Φ­(NO_2_^–^) = 0.054, Φ­(ONOO^–^) = 0.27) at 222 nm.^18^ In contrast, UV254 photolysis of nitrate is inefficient due to nitrate’s low molar absorptivity at this wavelength (ε ≈ 8 M^–1^ cm^–1^) rendering nitrate largely inert under conventional UV treatment conditions.^19^ Water matrix constituents, including nitrate, influence the performance of UV-based disinfection processes through mechanisms such as photon attenuation, radical scavenging, and sensitization. Compared to LP UV, Far-UVC processes at 222 nm are more susceptible to water matrix constituents, with nitrate in particular exerting a greater impact at 222 nm due to its higher absorbance and reactivity at shorter wavelengths.

While several recent studies have demonstrated that nitrate can enhance photolysis and advanced oxidation of chemical micropollutant contamination under 222 nm irradiation,^18,20,21^ it remains unclear whether these mechanisms contribute directly to microbial disinfection. Li et al. (2024) reported a 2.2-fold increase in sulfamethoxazole degradation with nitrate under UV222 irradiation, compared to UV222 alone, with RNS contributing up to 25% of the observed degradation at neutral pH.^20^ Yin et al. (2024) demonstrated that the UV222/nitrate process not only enhances photolysis of pesticides like atrazine and DEET but also generates RNS that induce nitration and nitrosation, leading to further degradation and formation of byproducts such as nitrophenols.^18^ Previous studies have shown that UV222 enhances the photolysis of organic micropollutants compared to conventional UV254.^21^ In addition, nitrate can act as an in situ oxidant under UV222, generating reactive species that enhance contaminant degradation at environmentally relevant concentrations (∼5 mg-N/L).^15^ Microorganisms differ fundamentally from chemical contaminants; varying structural characteristics consisting of capsid proteins, lipid membranes, and nucleic acids are targets for direct photolysis under 222 nm irradiation, and thus the balance between light-screening by nitrate and radical generation becomes critically important. Indeed, a recent study by Wang et al. (2023) found that nitrate inhibited 222 nm inactivation of *E. coli* due to photon competition and screening effects, revealing pathogen-specific matrix effects that warrant further study. However, current knowledge on the effects of NO_3_^–^ in Far-UVC systems on microbial disinfection is quite limited, particularly the role of nitrate in enhancing microorganism inactivation via radical production. For instance, Wang et al. (2023) observed that nitrate at environmentally relevant concentrations (0.5–10 mg-N/L) inhibits *E. coli* inactivation under Far-UVC by prolonging the so-called “lag phase” and reducing the observed inactivation rate constants by a factor of 1.08 to 2.74, an effect the authors attributed primarily to nitrate’s strong UV light–shielding effect.^22^ This finding underscores that nitrate’s impact under 222 nm is not universally beneficial: even when radical generation is possible, its photon screening effects may dominate, highlighting the need for mechanistic studies on bacteria and viruses.

Recent interest in Far-UVC degradation of chemical contaminants such as NDMA, carbamazepine and sulfamethoxazole,^14,20^ and the testing of KrCl* excimer lamps in field studies^23–25^ motivated an investigation of the potential benefits of KrCl* excimer lamps for disinfection via combined photon and oxidation pathways. While the role of nitrate-driven radicals has been widely explored in the degradation of organic micropollutants, these findings cannot be directly extrapolated to microbial systems. Radical reactivity with biological targets is constrained by diffusion, steric accessibility, and protective protein matrices, leading to fundamentally different inactivation pathways, particularly under simultaneous direct photolysis and oxidative attack.^26^ Wang et al. (2022) demonstrated that UVA irradiation (365 nm) in the presence of nitrite significantly enhanced microbial inactivation, achieving over 3-log reduction of *E. coli*, *S. aureus*, and *Spingopyxis sp.* compared to negligible effects from UVA or nitrite alone. The study attributed this enhancement primarily to reactive nitrogen species (NO_2_• and ONOO^–^/HOONO), which dominated over ROS in driving cell membrane disruption and DNA destruction.^27^

This study investigates the inactivation kinetics of two unique viruses, MS2 and T1UV bacteriophage, and a bacteria, *P. aeruginosa* under direct photolysis with and without nitrate present, using both UV254 and UV222 emitting lamps. Specifically, this work examines virus and bacteria UV-inactivation kinetics and elucidates the photochemical pathways underlying the observed kinetics of disinfection. By leveraging recent findings on nitrate quantum yields and reactive species formation, this research provides mechanistic insights into the efficacy of UV222/NO_3_^–^ driven oxidation for advanced water treatment applications and quantifies apparent biomolecular rate constants (*k*_app_) for both •OH and RNS inactivation of microorganisms that can be used to model oxidant-driven disinfection.

## 2 Materials and Methods

### 2.1 Microbial Methods

The inactivation of microorganisms during UV irradiation in the presence of nitrate, UV/NO_3_^–^, was assessed using three different model organisms.

MS2 bacteriophages (ATCC 15597-B1) were propagated and quantified using *E. coli* F_amp_ (ATCC 700891) as the host. Similarly, the T1UV bacteriophage (Laval University, HER#468) was propagated using *E. coli* CN13 (ATCC 700609). Enumeration of bacteriophages was conducted using the standard double agar layer method.^28,29^ For each dilution of each sample, a minimum of three replicate plates were prepared and enumerated. Briefly, 0.1 mL sample was combined with 0.1 mL of logarithmic phase *E. coli* host, and the resulting phage-host mixture was added to 5 mL of semisolid tryptic soy broth (TSB) (Difco, USA) amended with 0.075% (w/v) agar that was kept at 50 °C. After vortexing, the mixture was poured onto solid tryptic soy agar plates (Difco, USA) and allowed to solidify. Following overnight incubation at 37 °C, bacteriophages plaques were counted and recorded as PFU/mL.^29^ Frozen bacteriophage stocks of 10^10^ PFU/mL were diluted to 10^7^ PFU/mL in the irradiation solution with continuous stirring for UV irradiation. Detailed information about bacteriophage stock preparation is provided in the Supporting Information Text S1.

*Pseudomonas aeruginosa* (ATCC 15442) was employed as a model bacteria in pure culture experiments due to its relevance as an opportunistic waterborne pathogen in engineered water systems, including drinking water, aquaculture and reclaimed water.^30,31^ Exponential phase culture was grown in sterile TSB (BD Difco). Cultures were initiated from frozen stocks preserved in 20% (v/v) glycerol. Once the cultures reached exponential growth, bacterial cells were harvested by centrifugation (5,000 rpm, 10 min). The supernatant was discarded, and the cell pellet was resuspended in sterile phosphate-buffered saline (PBS) through vortexing. This washing step was repeated three times to effectively remove any residual growth media from the bacterial suspension. This bacterial suspension was 10-fold serially diluted in the irradiation solution to achieve a concentration of 10^6^ CFU/mL. Enumeration of *P. aeruginosa* was performed using the drop plate method,^32^ with 10 or 100 μL of a sample that had been serially diluted in PBS, plated onto TSB agar (in triplicate). The plates were inverted and incubated at 37 °C for 16–20 h. Bacteria density was calculated by counting the number of *Pseudomonas aeruginosa* colonies and correcting for any dilution factors.

### 2.2 UV Exposure Experiments

UV lamps were set up in a bench-scale collimated beam apparatus. Two types of lamps were investigated during this study: an unfiltered KrCl* excimer lamp emitting primarily at 222 nm (UV222) (USHIO, Cypress, CA), and conventional LP mercury lamps emitting at 254 nm (UV254) (15W, G15T8, USHIO). The KrCl* excimer emits a narrow peak primarily at 222 nm with a small additional emission feature peaking at 258 nm. The emission spectra (Figure 1A) of UV devices were measured using a calibrated Maya 2000 Pro spectrometer (Ocean Insight, Dunedin, FL). Incident UV irradiance was measured using a calibrated radiometer and sensor (models ILT2400 and SED240, respectively International Light Inc., Peabody, MA, USA), with values of (mean ± *S*.D): LP UV = 1.472 ± 0.021 mW/cm^2^ and UV222 = 0.623 ± 0.093 mW/cm^2^. The average irradiance was determined by correcting the incident irradiance for the water factor, reflection factor, divergence factor, and Petri factor. UV fluence (mJ/cm^2^) was calculated according to the Bolton-Linden standard method^33^ where the exposure time (sec) is multiplied by the calculated average irradiance (mW/cm^2^).

**Figure 1:**
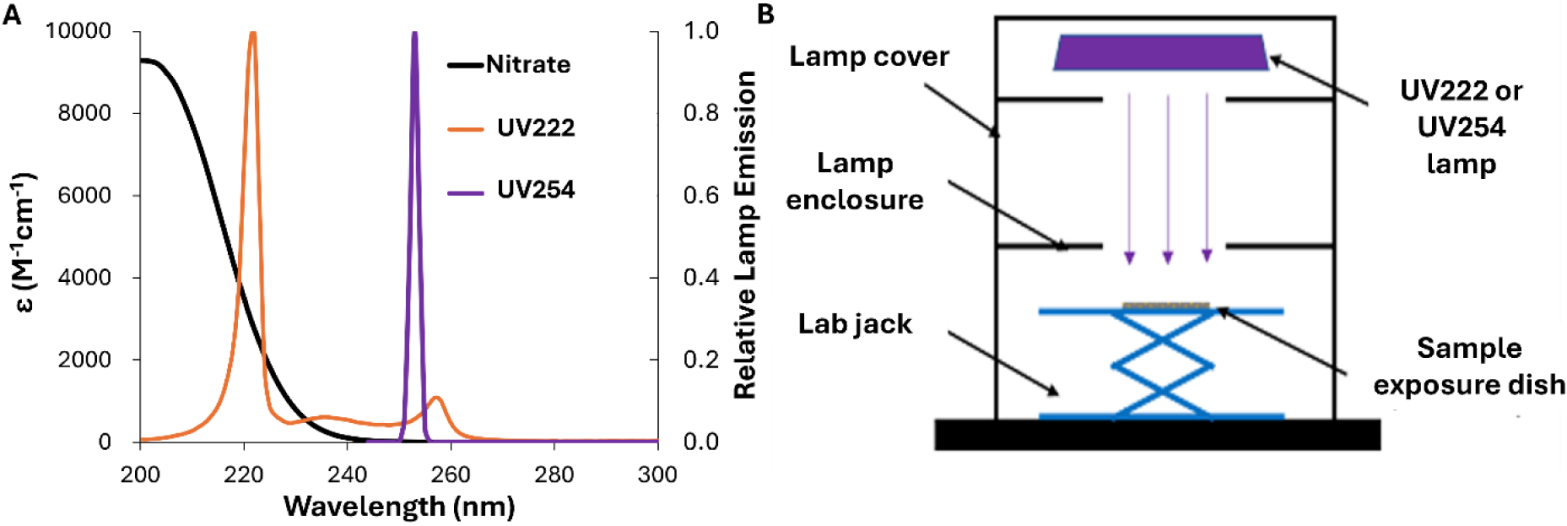
(A) Molar absorption spectra of nitrate and emission spectra of UV222 and UV254 sources used in this study (B) experimental set up.

UV/NO_3_^–^ exposure experiments were conducted using 5 mL of 10^6^ CFU-PFU/mL microorganisms added to a 33 × 20 mm (diameter × height) round crystallization dish (0.59 cm solution depth) with a small stir bar to induce good mixing without disturbing the water surface (Figure 1B). The UV lamps were warmed up to stabilize before use (i.e., 30 min for UV254 lamp and 5 min for UV222). The dish was placed 22.4 and 10 cm beneath the LP UV and unfiltered KrCl* excimer lamp, respectively. All samples after UV exposures were collected sacrificially, so no subsampling was performed in the UV exposure tests. All collected samples were covered with aluminum foil and kept in the dark to avoid photorepair of UV-induced genome damage while awaiting plating. Triplicate samples were irradiated for all data points. Control samples (without UV irradiation) were performed for all the irradiation conditions and collected in triplicate at both the start of the irradiation procedure (time 0) and at the longest exposure time. This was done to ensure that no natural microorganism decay occurred during the experimental period. In the UV222/NO_3_^–^ system different conditions were examined; NO_3_^–^ at concentrations of 4 and 8 mg-N/L were tested, as these represent environmentally relevant concentrations (U.S. EPA maximum contaminant level is 10 mg-N/L).

Tert-butyl alcohol (TBA) was used to quench hydroxyl radicals, to determine whether •OH played a significant role in microorganism inactivation. Tert-butyl alcohol absorbs UV negligibly at 222 nm (ε = 0.097 M^–1^ cm^–1^) and reacts with •OH (*k* = 6.0 × 10^8^ M^–1^ s^–1^).^34^ In these experiments, TBA was used at 200 mM to sufficiently quench •OH. A preliminary quenching experiment was conducted at TBA concentrations of 0 mM, 100 mM, 200 mM and 300 mM (Figure S1, Supporting Information); because no additional inactivation suppression was observed beyond 200 mM, a concentration of 200 mM TBA was chosen for the main experiments. Previous research demonstrated that unfiltered KrCl* excimer irradiation of TBA at 300 mM does not generate reactive oxygen species such as •OH and superoxide (O_2_^·–^) which could interfere with evaluation of microorganism inactivation.^20^ Uric acid was used as a quencher for reactive nitrogen species. Although uric acid exhibits UV absorption at 222 nm, the concentrations used in our quenching experiments (nitrate 4 mg-N/L + TBA 300 mM + UC 800 μM) result in minimal absorbance in the reaction medium. The absorbance with uric acid (nitrate 4 mg-N/L + TBA 300 mM) was 0.596 cm^–1^ and without it was 0.533 cm^–1^. Such a small inner-filter effect is negligible compared to the biological responses measured. While the mechanism of uric acid as a quencher of RNS is complex, uric acid reacts directly with peroxynitrous acid (HONOO) at a rate of 155 M^–1^ s^–1^ at 25 °C.^35,36^ Furthermore, uric acid likely reacts with the byproducts of the reaction between peroxynitrite (ONOO^–^) and CO_2_, present in aerated solutions. Uric acid reacts with •OH so TBA was used in combination with uric acid to ensure that uric acid reacted primarily with reactive nitrogen species, namely peroxynitrite, ONOO^–^. Uric acid also reacts with NO_2_ (1.8 × 10^7^ M^–1^ s^–1^), which is also produced from nitrate photolysis as well as from the reaction between nitrite and •OH (1 × 10^10^ M^–1^ s^–1^). While TBA addition will prevent the formation of NO_2_ via the reaction between nitrite and •OH, NO_2_ will still be produced from direct nitrate photolysis reactions. Therefore, in practice, uric acid addition represents nonselective quenching of reactive nitrogen species (RNS). Peroxynitrite and •OH are produced via distinct pathways, so •OH quenching by TBA will not significantly influence ONOO^–^ formation.^37^ Although UA is not a perfectly selective quencher and reacts slowly with ONOO^–^ (155 M^–1^ s^–1^),^35^ our calculations indicate that NO_2_• reacts with UA more than an order of magnitude faster than with microbial targets under the tested conditions (800 μM UA, MS2 = 10^7^ PFU mL^–1^) (Text S2, Supporting Information). Consistent with this, inactivation in the presence of UA returns to baseline levels without nitrate (demonstrated in Section 3.2 and Figure S5, Supporting Information), demonstrating that the UA concentration used is sufficient to effectively suppress RNS contributions in this system. Irradiation solutions for UV/NO_3_^–^ exposure were added to microorganism suspensions immediately prior to UV irradiation, and irradiation was initiated without an extended preincubation period. All UV/NO_3_^–^ experimental conditions are detailed in Table S1, Supporting Information.

### 2.3 Data Analysis

Log reduction value (LRV) was calculated using eq 1:

1 $$LRV=\mathrm{Log}\left(\frac{N_{0}}{N_{t}}\right)$$

Where N_0_ is the number of colony- or plaque-forming units (CFU or PFU/ml) of the unirradiated control, and N_t_ is the CFU or PFU/ml for each sample at irradiation time t.

The UV fluence-response data in this study for MS2 and T1UV were evaluated using the linear model, eq 2, described in^38^

2 $$\frac{N_{t}}{N_{0}}=10^{-kF_{N}}$$

Where F_N_ is the UV fluence (mJ/cm^2^) required to achieve N-log inactivation, and *k* (cm^2^/mJ) is the inactivation rate constant.

For the Weibull model applied to bacterial inactivation under UV irradiation, the LRV is described in eq 3:^39^

3 $$\log\left(\frac{N_{t}}{N_{0}}\right)={\left(\frac{F_{N}}{\delta }\right)}^{p}$$

Where F_N_ is the UV fluence (mJ/cm^2^), δ is the scale parameter, representing the UV fluence required to achieve a 1-log reduction when *p* = 1, and *p* is the shape parameter, which reflects the curvature of the inactivation kinetics. If *p* = 1, the model simplifies to a first-order (log-linear) inactivation process. If *p* < 1 (concave downward), the model exhibits tailing behavior, indicating that a fraction of the microbial population becomes less susceptible to UV exposure due to factors such as aggregation or light screening^40–42^ from UV exposure over time or UV fluence. If *p* > 1 (concave upward), the model reflects shouldering, suggesting initial resistance followed by accelerated inactivation as damage accumulates in the cells.

Paired *t* test were used to determine if there was a significant difference between two paired results (e.g., the LRV achieved at a given UV fluence between different exposure conditions and wavelengths).

### 2.4 Materials and Reagents

All stock solutions and reagents used in this study were prepared in ultrapure water (resistance of 18.2 MΩ cm). Tert-butyl alcohol (99.5%) and sodium nitrate (>99%) were purchased from Acros Organics, Fisher Scientific (Waltham, MA). Uric acid (>99%) was purchased from Sigma-Aldrich (St. Louis, MO).

### 2.5 Analytical Methods

Nitrate was measured using HACH TNT835 test vials and nitrite was measured using HACH TNT839 in a HACH DR6000 spectrophotometer. UV absorbance was measured in a 1 cm quartz cuvette using a Cary 4000 UV–visible spectrophotometer with baseline correction. An Agilent 1200 series HPLC instrument equipped with a UV detector and a reverse phase C-18 column (all from Agilent, Santa Clara, CA) was used to analyze para-chlorobenzoic acid (pCBA). pCBA was eluted with 10 mM phosphoric acid (pH ∼ 2.5) and methanol [45:55 (v/v)] using 234 nm for absorbance detection.

### 2.6 Apparent Biomolecular Rate Constants

pCBA was chosen as a probe compound due to its fast reaction with hydroxyl radicals (*k*•_OH/pCBA_ = 5 × 10^9^ M^–1^ s^–1^),^43^ its known direct photolysis rate at 222 nm, and likely low reactivity with RNS due to its electron-withdrawing groups.^15^ The pseudo-first-order rate constant (*k*′) of pCBA degradation was determined by plotting the natural logarithm of the ratio of the final (*C*) and initial (*C*_0_) pCBA concentrations as a function of the average UV fluence (*F*_avg_), as shown in eq 4

4 $$k^{\prime }=\frac{\ln\left(\frac{\left[pCBA\right]}{{[pCBA]}_{0}}\right)}{F_{avg}}$$

The steady-state concentration of hydroxyl radicals was then calculated. by solving eq 5 for [•OH]_ss_.^44^ The pCBA decay due to indirect photolysis was calculated by subtracting the direct photolysis rate of pCBA (on an average fluence basis), measured as 0.00136 cm^2^/mJ for UV222.

5 $$\ln\left(\frac{\left[pCBA\right]}{{[pCBA]}_{0}}\right)=\frac{-k_{•\mathrm{OH}/\mathrm{pCBA}}}{E_{avg}}{[•\mathrm{OH}]}_{ss}F_{avg}$$

To enable mechanistic comparison of radical-mediated inactivation across microorganisms, apparent bimolecular rate constants (*k*_app_) were derived. Unlike fluence-based inactivation constants (*k*), which capture the combined effects of direct photolysis and indirect oxidation, *k*_app_ isolates the effective reactivity between a given radical species and a microbial target. This approach allows evaluation of organism-specific susceptibility to radical attack independent of radical production rates. The contribution of total radicals (•OH + RNS) in microbial inactivation was calculated using eq 6

6 $$\Delta k_{\mathrm{total}\mathrm{radicals}}=k_{\mathrm{with}NO_{3}^{-}-}k_{direct}$$

Where $k_{\mathrm{with}NO_{3}^{-}}$ is the inactivation rate constant (cm^2^/mJ) from UV222/NO_3_^–^ condition, and *k_direct_* is the inactivation rate constant (cm^2^/mJ) from direct UV222 condition.

The contribution of hydroxyl radical in microbial inactivation was calculated using eq 7

7 $$\Delta k_{•\mathrm{OH}}=k_{\mathrm{with}NO_{3}^{-}}-k_{NO_{3}^{-}+TBA}$$

Where $k_{NO_{3}^{-}+TBA}$ is the inactivation rate constant (cm^2^/mJ) from UV222/NO_3_^–^ with TBA condition.

The contribution of RNS in microbial inactivation was calculated using eq 8

8 $$\Delta k_{\mathrm{RNS}}=k_{NO_{3}^{-}+TBA}-k_{NO_{3}^{-}+TBA+UA}$$

Where $k_{NO_{3}^{-}+TBA+UA}$ is the inactivation rate constant (cm^2^/mJ) from UV222/NO_3_^–^ with TBA and uric acid (UA) condition. All *k* values are the inactivation rate constant (cm^2^/mJ) from fluence-response data under different conditions. *k* and Δ*k* values are presented in Table S2, Supporting Information. This method assumes that direct photolysis and radical-mediated pathways contribute additively to overall inactivation, and that scavenger addition selectively suppresses targeted radical pathways without altering direct UV damage.

Apparent molecular rate constants (*k*_app_) were calculated between a radical species (•OH or RNS) and each microorganism (virus or bacterium). These reported rate constants reflect the overall net reactivity of the radical with the organism inclusive of all possible molecular targets (capsid proteins, nucleic acids, membranes, etc.) leading to inactivation as condition-specific, apparent descriptors of organism susceptibility rather than intrinsic molecular rate constants. The calculation of *k*_app_ was facilitated by kinetic modeling, to predict the degradation rate of a contaminant (in this case a microorganism) without needing to track the short-lived hydroxyl radical directly.

The *k*_app_ for hydroxyl radical species was calculated using eq 9

9 $$k_{app}\left(•O\mathrm{H,}\mathrm{organism}\right)=\frac{\Delta k_{•\mathrm{OH}}}{{[•OH]}_{SS}}$$

Where Δ*k* was calculated using eq 7 and [•OH]_SS_ was determined by kinetic modeling and validated experimentally in eq 5 The *k*_app_ for reactive nitrogen species was calculated using eq 10:

10 $$k_{app}\left(\mathrm{RNS,}\mathrm{organism}\right)=\frac{\Delta k_{\mathrm{RNS}}}{{[\mathrm{RNS}]}_{SS}}$$

While [RNS]_SS_ was determined by kinetic modeling, numerous different RNS are generated simultaneously under UV222 (ONOO^–^, NO_2_•, NO•, N_2_O_3_, etc.) and cannot be selectively quenched due to the lack of radical quenchers which are specific to one RNS but not others. Hence RNS were treated collectively as an effective oxidative pool capturing the cumulative reactivity of RNS generated under UV222. Therefore, for this analysis the contribution of RNS is grouped together to represent the cumulative RNS reactivity. As a result, the *k*_app_ values calculated are specific to the conditions studied here (pH, background water matrix, nitrate concentration, lamp irradiance, etc.), as RNS speciation may change with different initial conditions.

### 2.7 Kinetic Modeling

A kinetic model for UV222-driven radical formation was developed using Kintecus.^45–47^ In short, the model included 182 reactions for radical generation from nitrate photolysis and interactions between phosphate/chloride and reactive species. The nitrate photolysis model was adapted from Yin et al. (2024) and reactions involving chloride, which is introduced from the PBS matrix, were added.^18,46,47^

## 3 Results and Discussion

### 3.1 Bacteriophage Direct Photolysis under UV222 and UV254 Based UV/NO_3_^–^

#### 3.1.1 MS2 Inactivation

Inactivation of MS2 bacteriophage by direct photolysis and UV/NO_3_^–^ were investigated in buffered lab water under both UV254 (Figure 2A) and UV222 (Figure 2B). MS2 inactivation significantly improved under UV222 direct photolysis and UV222/NO_3_^–^, compared to UV254, as evidenced by up to an order-of-magnitude increase in the inactivation rate constant for UV222 (Table S2, Supporting Information). Hull and Linden (2018) observed that MS2 exhibits greater sensitivity to direct photolysis under UV222 than UV254 with two times higher *K* value for UV222,^10^ similar to what was observed in this study for direct photolysis. The enhanced direct inactivation by UV222, compared to UV254, is attributed to additional damage mechanisms, including protein denaturation. While LP UV primarily induces damage through the formation of cyclobutane pyrimidine dimers (CPD) and other photochemical lesions in DNA/RNA, which inhibit replication,^7,48^ KrCl* excimer irradiation delivers a more comprehensive attack on viruses. In addition to generating similar nucleic acid photoproducts, the higher energy 222 nm photons are readily absorbed by proteins, causing direct damage to viral capsid proteins and even lipid membranes,^7,49^ impacting the virus infection cycle. When comparing the UV222 direct photolysis to UV222/NO_3_^–^ system, at a UV fluence of 10 mJ/cm^2^, UV222 direct photolysis resulted in an average LRV of 1.41 ± 0.12, whereas the addition of nitrate at concentrations of 4 and 8 mg-N/L significantly enhanced inactivation, yielding LRVs of 2.38 ± 0.06 and 2.27 ± 0.18, respectively (*P* < 0.05, paired *t* test). For UV254, the presence of NO_3_^–^ did not significantly affect the inactivation kinetics of MS2 compared to direct UV photolysis without NO_3_^–^, due to the limited absorbance of nitrate at 254 nm. The MS2 inactivation rate constant is approximately 5–7 times faster for UV222/NO_3_^–^ system than UV254/NO_3_^–^ system. The enhancement for UV222/NO_3_^–^ system is attributable to the photochemical generation of reactive species, notably hydroxyl radicals (•OH) and RNS, such as nitrogen dioxide (NO_2_•) and peroxynitrite (ONOO^–^). MS2 reacts rapidly with •OH (*k* = 10^11^ M^–1^ s^–1^), which is minimally produced under 254 nm irradiation compared to 222 nm irradiation (Figure S2 and Table S3, Supporting Information). This is consistent with previous research showing that nitrate presence accelerates contaminant degradation.^18,20,50^ While the 4 and 8 mg/L nitrate concentration produces additional •OH, it also produces more nitrite, and •OH scavenging by nitrite can lower the steady state concentration of •OH, decreasing the benefit to MS2 inactivation. Such competing interactions between nitrate/nitrite photochemistry and radical scavenging have been shown to affect radical yields in Far-UVC 222 nm systems, where nitrate/nitrite absorb strongly at 222 nm and influence both light screening and reactive species formation.^51^ Therefore, it was hypothesized that nitrite formation during the production of radicals from nitrate photolysis could partially neutralize the activity of reactive oxygen species contributing to UV disinfection, limiting indirect photolysis disinfection mechanisms. To evaluate the potential role of nitrite as a secondary scavenger of hydroxyl radicals, nitrite formation was quantified during UV222 irradiation in the presence of 4 mg-N/L nitrate and MS2 bacteriophage (Figure S3, Supporting Information). Nitrite concentrations increased with UV fluence, reaching 0.16 mg-N/L at 25 mJ/cm^2^. Despite this, the combination of direct photolysis (targeting both RNA and protein structures) and oxidative damage induced by nitrate photolysis significantly enhances MS2 bacteriophage disinfection. Future work will be needed to evaluate higher nitrate concentrations where competing scavenging pathways may limit or alter the observed benefit.

**Figure 2:**
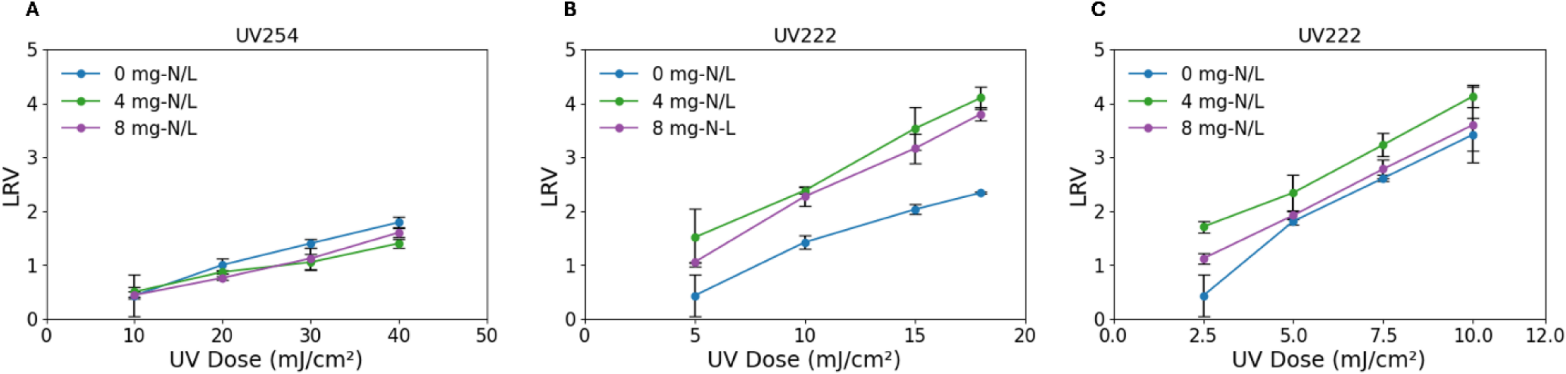
Inactivation of (A) MS2 bacteriophage under UV254 and (B) MS2 bacteriophage under UV222 (C) T1UV bacteriophage under UV222 at varying nitrate concentrations: 0, 4, and 8 mg-N/L (legend refers to nitrate concentration) in buffered lab water. Log reduction value (LRV) was calculated using eq 1, modeled fitted using eq 2. Error bars represent one standard deviation of the means (*n* = 3). All UV doses were calculated using average irradiance which includes the water factor, reflectance factor, Petri factor and divergence factor.

#### 3.1.2 T1UV Inactivation

To explore the efficacy of UV222/NO_3_^–^ system on inactivation of viruses with different physical and genomic structures, the inactivation of T1UV was also investigated (Figure 2C). While MS2 represents a small, single-stranded RNA (ssRNA) bacteriophage with a simple protein capsid, T1UV is a larger genome double-stranded DNA (dsDNA) bacteriophage with a complex capsid (Table 1). At a UV fluence of 10 mJ/cm^2^, direct photolysis at UV222 resulted in an average LRV of 3.42 ± 0.31. The addition of nitrate at concentrations of 4 and 8 mg-N/L slightly enhanced inactivation, yielding LRVs of 4.11 ± 0.21 and 3.68 ± 0.51, respectively. While a trend toward increased inactivation was observed at 4 mg-N/L, paired *t* tests across conditions showed no statistically significant differences across different nitrate concentrations (*P* > 0.05, paired *t* test). The sensitivity of direct photolysis under unfiltered KrCl* excimer irradiation aligns with the findings of Ma et al. (2022), who observed that T1UV is highly susceptible to UV222. While Ma reported a higher inactivation rate constant of 0.70 ± 0.01 cm^2^/mJ using a filtered KrCl* excimer (emission at wavelengths longer than 222 nm was filtered out), our study using an unfiltered source (∼75% of the emission is at 222 nm) yielding a lower rate constant (0.35 cm^2^/mJ).^52^ In the context of direct photolysis using UV222, the T1UV bacteriophage demonstrates higher intrinsic sensitivity than MS2 (Table S2 and S4, Supporting Information), achieving a greater inactivation rate constant and log reduction under identical UV fluence levels.

**Table 1:** Characteristics of Viruses Tested in This Study

				Genome features			
Virus	ATCC #	Host cell	Family	Size	Type	Capsid shape and size	Capsid structure
MS2	15 597-B1	*E. coli* *F*_amp_	*Leviviridae*	3.6 Kb	ssRNA(+)	Small icosahedral (no tail)	180 identical subunits + A-protein
						27 nm in diameter (Strauss and Sinsheimer)	
T1UV	N/A^*‡*^	*E. coli* *CN13*	*Siphoviridae*	48.8 Kb	dsDNA	Large icosahedral head + long tail	Multiple proteins
						60 nm head, with 150 nm long tail	

The contrasting nitrate effect between MS2 and T1UV is more precisely interpreted as a balance between (i) the efficacy of direct 222 nm photolysis and (ii) the accessibility of oxidation-susceptible viral targets to nitrate-derived radicals. When direct photolysis alone rapidly compromises infectivity, as observed for T1UV, additional oxidative pathways are relatively less important. In contrast, when direct photolysis is less dominant, as for MS2, oxidative damage can contribute measurably to overall inactivation. Table 1 compares the key structural and genomic characteristics of MS2 and T1UV. The high susceptibility of T1UV (compared to MS2) under UV222 is likely related to direct photolysis damage; its large dsDNA genome, structural complexity of a larger icosahedral head (∼60 nm) filled with dsDNA, surrounded by accessory proteins, and coupled to a long noncontractile tail, may offer weak spots for UV-induced conformational damage.^53^ MS2 on the other hand, possesses a small, ssRNA genome that, although inherently capable of absorbing UV radiation, tends to form fewer UV-induced lesions that block replication, and experiments show that RNA damage mirrors infectivity loss.^7^ This resistance of MS2 to severe genomic lesioning is further enhanced by its tightly packed icosahedral capsid, composed of 180 homologous coat protein subunits and the maturation protein (A-protein), optimized to protect its RNA and even bind the genome to nucleate assembly, increasing resilience.^54,55^ Unlike UV254, previous studies suggested that the UV sensitivity of viruses at 222 nm is not only affected by genome makeup, but also by the enhanced protein absorption at shorter wavelengths.^52^

Interestingly, when nitrate is present at 4 or 8 mg-N/L, the disinfection rate of T1UV remained largely unchanged compared to the absence of nitrate. The high intrinsic sensitivity of T1UV to direct photolysis under UV222 likely dominates the inactivation kinetics and may decrease the relative additional contributions from oxidative pathways under the tested conditions. The more complex, tailed virion structure of T1UV may further restrict access to oxidation-susceptible targets, rendering genomic DNA damage the primary determinant of infectivity loss which has already been efficiently induced by direct 222 nm photolysis^56,57^ . In contrast, MS2 displayed a significant enhancement under the same conditions. Prior studies confirm that •OH preferentially inactivate ssRNA viruses like MS2 through capsid oxidation and RNA strand breakage, whereas dsDNA viruses such as T1UV are less impacted by oxidation.^58,59^ The relatively rapid UV222 direct photolysis of T1UV likely also reduces the effective exposure time of that virus to hydroxyl radicals during the experiments, such that even if radicals are produced, their proportional contribution to total inactivation is diminished compared with MS2. For example, to achieve approximately 4 LRV, MS2 required about 64 s under direct photolysis, which decreased to 36 s in the presence of 4 mg-N/L nitrate (under identical UV fluence rates) whereas T1UV reached similar inactivation levels in only 18–19 s under the same conditions. In other words, when direct UV damage dominates the early kinetics, additional oxidative pathways may have less opportunity to contribute measurably to overall inactivation. For MS2, the single A-protein are primary targets of singlet oxygen (^1^O_2_) and other ROS. Oxidation at these sites leads to capsid aggregation and protein cross-linking, causing loss of antigenicity and infectivity.^60^ Moreover, specific amino acids such as histidine, tryptophan, methionine, cysteine, and tyrosine, which are particularly susceptible to oxidation, are located in antigenic sites on the A- protein. Oxidative damage to these residues, especially histidine and tryptophan within critical antigenic determinants, accelerates the loss of infectivity through structural compromise of the capsid.^54,55,61^

### 3.2 Contribution of Reactive Species to Inactivation in UV222/NO_3_^–^ System

Given the enhancement in MS2 inactivation observed in the UV222/NO_3_^–^ system, the role of nitrate-derived reactive species was investigated. To elucidate the role of hydroxyl radicals in MS2 inactivation, the radical scavenger TBA was used to quench hydroxyl radicals, preventing the reaction between •OH and MS2 (Figure 3, the entire UV fluence-response curve presented in Figure S1, Supporting Information). While TBA is commonly used to quench hydroxyl radicals, it is not perfectly selective; reactions such as TBA + •OH can yield minor byproducts including *tert*-butyl peroxyl and, under some conditions, superoxide.^15,62^ In systems with significant organic sensitizers, TBA can also interact with triplet states. However, in the nitrate-only Far-UVC system studied here, the primary effect of TBA is rapid •OH scavenging.^63,64^ At a UV fluence of 15 mJ/cm^2^, the LRV decreased ∼1 log in the presence of TBA, indicating that hydroxyl radicals contribute to MS2 inactivation. Dark control experiments demonstrated that no MS2 inactivation occurred from TBA directly absent of 222 nm irradiation (Figure S4, Supporting Information). These results indicate that the formation of •OH from nitrate photolysis (when not scavenged by TBA) enhances disinfection of MS2, likely via capsid oxidation and RNA strand breakage. TBA may also inadvertently quench any NO_2_• formed from the reaction between nitrite and •OH, and thus the contribution of NO_2_• to MS2 inactivation cannot be entirely ruled out using TBA quenching experiments. However, nitrite formation was minimal during UV222 exposure (0.0945 ± 0.0012 mg-N/L at a UV fluence of 15 mJ/cm^2^, Figure S3, Supporting Information), suggesting that NO_2_• generation via nitrite was unlikely to contribute significantly to disinfection (Figure S3, Supporting Information). Moreover, direct NO_2_• formation from nitrate photolysis is ∼10× faster than via •OH scavenging of nitrite under our conditions (Text S3, Supporting Information), indicating that secondary NO_2_• formation pathways are minor and that TBA’s influence on total NO_2_• production does not substantially impact the assessment of RNS contributions. Ultimately, NO_2_• generation via nitrite was unlikely to contribute significantly to disinfection.

**Figure 3:**
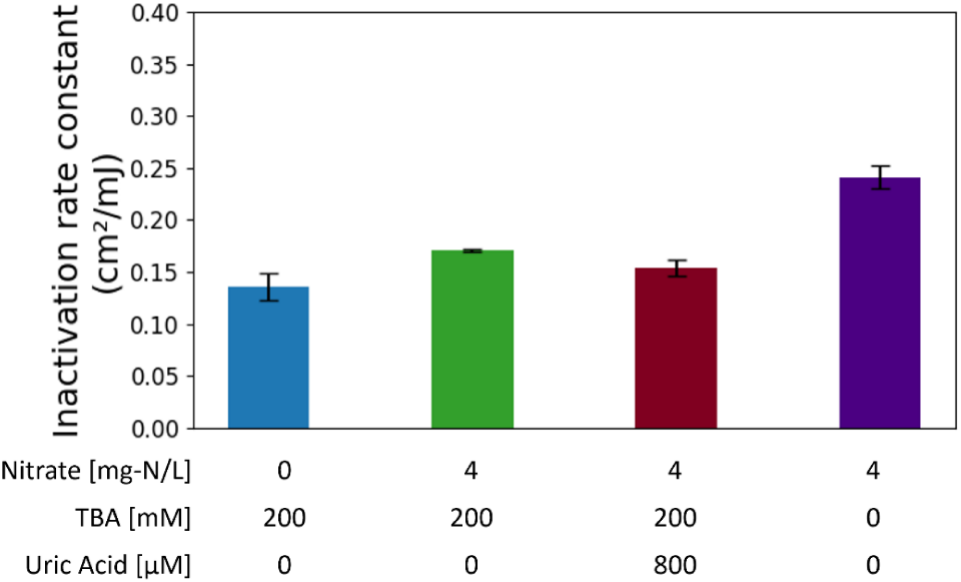
MS2 inactivation rate constant at different initial nitrate, uric acid and TBA concentrations (table refers to the concentrations). Error bars represent standard deviation of duplicate experiments (*n* = 2).

Previous studies also found that hydroxyl radicals play a significant role in MS2 bacteriophage inactivation. In a study of UV/Cl_2_, Rattanakul and Oguma (2017) demonstrated that •OH accounted for a significant portion of MS2 inactivation under combined UV254 nm and chlorine exposure.^65^ Similarly, Mamane et al. (2007) reported that supplementing filtered UV irradiation (>295 nm) with 25 mg/L H_2_O_2_ led to approximately 2.5-log enhanced MS2 inactivation, underscoring the relevant contribution of •OH-mediated oxidation in the process.^58^

To investigate the role of RNS in MS2 inactivation under UV222 in the presence of nitrate (4 mg-N/L), 800 μM uric acid was added as a known scavenger for NO_2_• and peroxynitrite, along with 200 mM TBA (Figure 3, the entire UV Fluence-response curve presented in Figure S5, Supporting Information). The experiments were performed only at a nitrate concentration of 4 mg-N/L because as indicated in Figure S5, there was no additional benefit to MS2 inactivation at a nitrate concentration of 8 vs 4 mg-N/L. The addition of uric acid exhibited approximately a 0.3-log lower inactivation at a UV fluence of 15 mJ/cm^2^ compared to without uric acid (*P* < 0.05, paired *t* test), both of which included 200 mM TBA to quench hydroxyl radicals. These results suggest that under these conditions, RNS such as NO_2_• and HONOO/ONOO^–^ (Table S3, Supporting Information) contribute only slightly to MS2 inactivation.

While hydroxyl radicals exhibit high reactivity and nonselective oxidation, allowing them to damage numerous viral components, including capsid proteins and nucleic acids^66,67^ RNS such as nitrogen dioxide (NO_2_•) and peroxynitrite (ONOO^–^), are considerably less effective for virus inactivation. This is largely attributed to the comparatively lower RNS oxidation potential, (one-electron reduction potentials of E^0^ of 2.72, 2.14, 1.40, 1.04 V for OH, HONOO, ONOO^–^, NO_2_, respectively),^68^ selective target preferences (e.g., aromatic amino acids),^69^ and potentially limited access to interior capsid regions due to structural or electrostatic constraints. Viral capsid protein shells often carry distinct surface charge distributions and present densely packed protein subunits, which can limit the diffusion or access of charged reactive species such as ONOO^–^.^70,71^ Experimental work by Sun et al. (2016) quantified radical-specific inactivation rates under UV/H_2_O_2_ and UV/peroxydisulfate (PDS) and demonstrated that the disinfection efficacy of •OH against MS2 was significantly higher, exceeding that of carbonate and sulfate radicals by orders of magnitude.^59^ These findings reinforce that other than direct UV photolysis, •OH is the primary radical responsible for effective MS2 disinfection in the presence of UV-driven oxidation.

### 3.3 *P. aeruginosa* Direct Photolysis under UV222/NO_3_^–^

UV disinfection under UV222 was investigated with and without NO_3_^–^ addition for *P. aeruginosa* across varying nitrate concentrations (0, 4, and 8 mg-N/L) (Figure 4). The UV fluence-response data were fitted using the Weibull inactivation model (eq 3), with R^2^ > 0.93, confirming the model fitting. All fitted shape parameters were less than unity (*p* < 1), indicating a concave-downward curvature typical of tailing behavior, and the corresponding scale parameters (δ) did not vary significantly among nitrate conditions. All the calculated parameters are presented in Supporting Information: Table S5.

**Figure 4:**
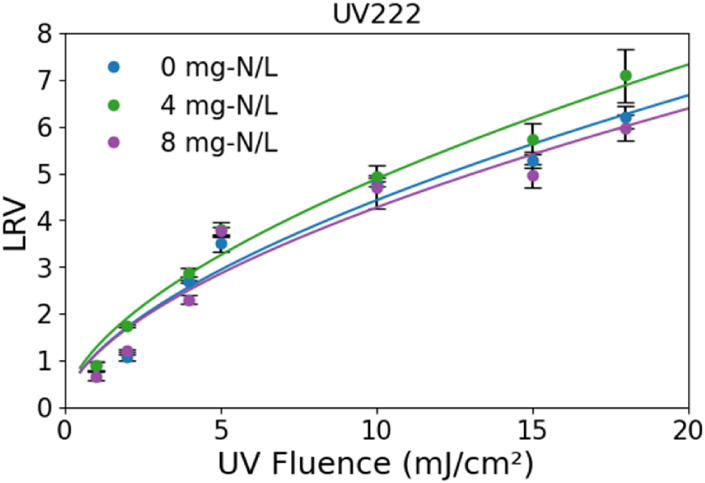
Inactivation of *P. aeruginosa* under unfiltered KrCl* excimer at varying nitrate concentrations: 0, 4, and 8 mg-N/L (legend refers to nitrate concentration) in buffered lab water. Log reduction value (LRV) was calculated using eq 1, modeled fitted using eq 3. Error bars represent one standard deviation of the means (*n* = 3). All UV doses were calculated using average irradiance which includes the water factor, reflectance factor, Petri factor and divergence factor.

The results of this study support and reinforce prior observations regarding the efficacy of UV222 disinfection for *P. aeruginosa*.^72,73^ Unlike MS2 and similar to T1UV, *P. aeruginosa* also exhibited minimal variation in disinfection performance with increasing nitrate concentration. UV222 without nitrate addition achieved an average LRV of 4.78 ± 0.06 after UV fluence of 10 mJ/cm^2^, while the addition of 4 and 8 mg-N/L nitrate yielded LRVs of 4.93 ± 0.03 and 4.70 ± 0.45, respectively (*P* > 0.05, paired *t* test). These results indicate that, for *P. aeruginosa* under UV222, inactivation is governed primarily by direct photodamage rather than by nitrate-driven oxidative pathways. One contributing factor may be the effective exposure time to hydroxyl radicals; *P. aeruginosa* reached high LRV more quickly under direct UV222 irradiation (Table S4, Supporting Information), which reduces the relative window for radicals to contribute additional damage. Bounty et al. (2012) examined adenovirus inactivation under a UV/H_2_O_2_ advanced oxidation process and reported that increased radical exposure correlates with higher viral loss, highlighting the importance of both radical concentration and exposure duration in AOP disinfection.^74^ The *P. aeruginosa* outcome aligns with Wang et al. (2023), who showed once the average UV fluence is corrected for nitrate’s absorbance, the nitrate has little to no positive effect on Far-UVC disinfection of *E. coli*. Both *E. coli* and *P. aeruginosa* are Gram-negative, rod-shaped bacteria but belong to different genera.^22^ Bacteria possess a complex outer membrane comprising lipopolysaccharides, membrane proteins, and peptidoglycan, which collectively serve as a physical barrier against reactive species,^69^ further limiting indirect radical damage during the short fluence required for direct UV222 photolysis. Beyond the direct DNA damage, at 222 nm irradiation, direct absorption by peptide bonds and aromatic amino acids in outer membrane proteins (and periplasmic enzymes) leads to rapid protein photolysis,^75^ compromising envelope integrity and repair systems much faster than radical attack alone would allow.^22,76^ Moreover, the bacterium’s cytoplasmic antioxidant systems, including catalase and superoxide dismutase (SOD), actively neutralize intracellular ROS, mitigating intracellular oxidative stress.^77^ Reactive species generated from nitrate photolysis did not result in any enhancement of inactivation of *P. aeruginosa*. Thus, the Gram-negative envelope can shield against •OH -mediated damage but is vulnerable to 222 nm. The limited influence of nitrate in this context highlights a key distinction in how bacteria and viruses (MS2, ssRNA virus) respond to radical-mediated disinfection. This divergence is rooted in fundamental structural and biochemical differences. MS2’s protein capsid and exposed RNA genome leave it highly vulnerable to oxidative damage from hydroxyl radicals (•OH) and nitrate-derived species (NO_2_•, ONOO^–^), as supported by studies such as Sun et al. (2016).^59^ The absence of enhanced inactivation despite nitrate addition suggests that the radicals formed under UV222/NO_3_^–^ system do not reach sufficient intracellular targets in *P. aeruginosa* or are scavenged before exerting an effect. Additionally, Li et al. (2024) noted that nitrate-derived species such as NO_2_• possess lower oxidative redox potential than hydroxyl radicals, potentially reducing their contribution to bacterial lethality.^78^ From a practical perspective, these findings demonstrate that for Gram-negative bacteria in UV222 systems, increasing nitrate concentration does not enhance disinfection efficacy, as direct UV-induced DNA damage and protein absorbance remains the dominant inactivation mechanism. In addition, higher nitrate concentrations can increase light attenuation in the water matrix, reducing the effective UV dose delivered to microorganisms.

### 3.4 Apparent Biomolecular Rate Constants for •OH and RNS with Microorganisms

To further quantify the role of radical pathways in disinfection, apparent biomolecular rate constants (*k*_app_) for •OH and RNS with MS2, T1UV, and *P. aeruginosa* were estimated (Table 2). Here, RNS refers to the bulk contribution of the many different species which are produced by nitrate photolysis (NO_2_•, ONOO^–^, etc.); as a result, for the analysis in this manuscript, the RNS concentration represents the sum of NO_2_• and ONOO^–^. Values used in the Kintecus kinetic model for 4 mg-N/L and 8 mg-N/L nitrate photolysis are presented in Supporting Information: Table S6 and Table S7, respectively. The reactions used in the Kintecus kinetic model are presented in Supporting Information: Table S8.

**Table 2:** Apparent Bimolecular Rate Constants (*k*_app_) for •OH and RNS with Organisms and Corresponding Calculation Parameters

Organism	Nitrate concentration (mg-N/L)	Δ*k* (cm^2^/mJ)^a^	[•OH]_ss_ (M)^b^	[•OH]_ss_ (M)^c^	[RNS]_ss_ (M)^c^	*k*_app_ (•OH,organism) (M^–^ ·s^–1^)	*k*_app_ (RNS,organism) (M^–1^ ·s^–1^)
MS2	4	0.107	1.57 × 10^–12^	1.59 × 10^–12^	1.91 × 10^–7^	4.43 × 10^10^	8.79 × 10^4^
	8	0.081	1.71 × 10^–12^	1.88 × 10^–12^	1.14 × 10^–7^	2.38 × 10^10^	1.46 × 10^5^
T1UV	4	0.080	1.57 × 10^–12^	1.59 × 10^–12^	1.91 × 10^–7^	5.14 × 10^10^	Negligible
	8	0.028	1.71 × 10^–12^	1.88 × 10^–12^	1.14 × 10^–7^	1.60 × 10^10^	Negligible
*P. aeruginosa*	4, 8	No change vs direct	—			Negligible	Negligible

a Δ*k* (cm^2^/mJ) for total radicals calculated using eq 6 and presented in the table. Δ*k* (cm^2^/mJ) for [•OH] calculated using eq 7 and Δ*k* (cm^2^/mJ) for RNS calculated using eq 8 and presented in Table S2, Supporting Information.

b Experimental value.

c Modeling value.

First, experimental pCBA data were used to validate the kinetic model. Experimentally derived [•OH]_ss_ was calculated as 1.57 × 10^–12^ M for 4 mg-N/L nitrate and 1.71 × 10^–12^ M for 8 mg-N/L, which agrees closely with the modeled results of 1.59 × 10^–12^ M for 4 mg-N/L and 1.88 × 10^–12^ M for 8 mg-N/L (Supporting Information: Figures S6A and S7A, respectively). Because these [•OH]_ss_ values were generated in the absence of microbial contaminants, they do not directly include consumption by microbial components, but still provide a useful basis for comparing conditions and interpreting the relative role of •OH in inactivation. In these experiments, PBS was used as the primary matrix, introducing significant chloride into the system. The radical scavenging and promotion reactions involving chloride were included in the kinetic model and illustrated that reactive chlorine species contributions were minor (reactions 130–187 in Table S8, Supporting Information). To validate this minor influence of the PBS matrix, parallel experiments were conducted in dechlorinated tap water with nitrate concentrations of 0 mg-N/L and 4 mg-N/L, using the same initial MS2 concentration. Under UV222, at fluences of 15–30 mJ/cm^2^, the enhancement in inactivation ranged from ∼1.3 to ∼1.9 log (Figure S8, Supporting Information), confirming that the observed effect is not an artifact of the PBS matrix.

Next, the modeled [•OH]_ss_ was used to calculate *k*_app_(•OH) which reflect the overall net reactivity of •OH radical with MS2, using eq 9 and Table S2 Supporting Information data. The calculated *k*_app_(•OH) ranged from 2.38 × 10^10^ to 4.43 × 10^10^ M^–1^·s^–1^ for MS2. This rapid bimolecular rate constant is consistent with diffusion-limited reactions of •OH with biomolecules such as nucleic acids and proteins. These values also agree well with prior literature.^59,65^ While diffusion-controlled limits for small molecules reacting with •OH are often cited as ∼10^10^ M^–1^·s^–1^, the effective diffusion-controlled limit for MS2 is much higher (∼6.6 × 10^11^ M^–1^·s^–1^) owing to the virus’s relatively larger size and collision cross-section.^65^ Modeled RNS concentrations were then used to calculate *k*_app_ (RNS). The total combined concentration of NO_2_ and ONOO^–^ was used in this calculation, as experiments with the uric acid quencher can not disentangle the contribution of these two species; modeling results indicated that no other RNS was present at a sufficiently high (>10^–8^ M) concentration to contribute meaningfully to MS2 inactivation (Supporting Information: Figures S6B and S7B, respectively). In contrast to *k*_app_(•OH), *k*_app_(RNS) was approximately 8.79 × 10^4^ M^–1^·s^–1^ and 1.46 × 10^5^ M^–1^·s^–1^ for 4 and 8 mg-N/L respectively, nearly 5 orders of magnitude lower. The large difference in apparent bimolecular rate constants is consistent with the broad, nonselective reactivity of •OH with diverse biological macromolecules including DNA and proteins, leading to broad oxidative damage.^79^ In contrast, RNS, such as peroxynitrite and nitrogen dioxide, react more selectively with electron-rich functional groups such as phenols and anilines commonly found in aromatic amino acids like tyrosine and tryptophan present in the coat protein sequence,^80^ leading to less pervasive damage of microbial targets.^81–85^ These apparent molecular rate constants were derived using the modeling results for steady-state RNS concentrations which ranged from 1.91 × 10^–7^ M for 4 mg-N/L and 1.14 × 10^–7^ M for 8 mg-N/L.

It is important to recognize that the contribution of each radical species to inactivation depends not only on its apparent bimolecular rate constant (*k*_app_) but also on its steady-state concentration. For each radical, the overall radical-mediated inactivation rate can be expressed as eq 11:

11 $$\mathrm{The overall inactivation rate}=k_{app}\left(\mathrm{radical}\right)\times {[\mathrm{radical}]}_{ss}$$

The calculation was performed for hydroxyl radicals using *k*_app_(•OH) and the modeled steady-state [•OH]_ss_ and for RNS radicals using *k*_app_(RNS) and the modeled steady-state [RNS]_ss_, as indicated in eqs 9 and 10 respectively. Using this approach, the approximate overall inactivation rate for RNS is 10^–3^ s^–1^ (= 10^–8^ M × 10^5^ M^–1^·s^–1^), while for •OH it is 10^–2^ s^–1^ (= 10^–12^ M × 10^10^ M^–1^·s^–1^), meaning the overall •OH reaction rate is about 10 times higher than for RNS under those conditions. This quantitative calculation confirms that, for UV/NO_3_^–^ conditions examined here, and considering only radical-mediated pathways, •OH is the dominant radical driving MS2 inactivation, while RNS such as NO_2_• and ONOO^–^ provide only a marginal contribution. Thus, while •OH likely reacts with viruses at the diffusion-controlled limit, RNS reactivity is influenced by chemical selectivity, which reflects the electrophilic nature of RNS such as NO_2_•. For T1UV, *k*_app_(•OH) values were in the 1.60–5.14 × 10^10^ M^–1^·s^–1^ range which is comparable to MS2, and the total contribution of radicals to inactivation was modest due to the dominance of direct photolysis at UV222. This finding reinforces that T1UV inactivation is primarily governed by its intrinsic sensitivity to direct UVC damage, with radicals contributing little additional benefit. For *P. aeruginosa*, nitrate addition produced no measurable enhancement of inactivation, yielding negligible *k*_app_ values for both •OH and RNS. The lack of response is consistent with the protective role of the Gram-negative outer membrane, which limits radical penetration and damage.

Overall, these findings provide quantitative estimates of organism-specific *k*_app_ values for radicals generated under UV222/NO_3_^–^ conditions. They demonstrate that •OH is highly effective against viruses (particularly MS2), while RNS are comparatively less important, and bacteria such as *P. aeruginosa* remain largely resistant to both •OH and RNS. This analysis adds a mechanistic and predictive layer beyond previous qualitative observations for interpreting radical contributions under UV222/NO_3_^–^ conditions.

### 3.5 Environmental Implications

The use of 222 nm Far-UVC irradiation coupled with nitrate-driven radical formation presents a promising strategy for addressing microbial and chemical contaminants in diverse environmental water matrices, including wastewater effluent, surface runoff, and agricultural drainage, and aquaculture, where nitrate is naturally present. This indigenous nitrate can serve as a precursor for in situ generation of ROS and RNS, particularly under Far-UVC light where nitrate absorbs strongly, thereby reducing the need for chemical additives like hydrogen peroxide and potentially lowering operational costs and environmental burden. Recent works have shown that 222 nm irradiation substantially enhances chemical degradation such as for N-nitrosodimethylamine (NDMA) and carbamazepine (CBZ) via direct photolysis or radical-mediated mechanisms (ROS+RNS), respectively, compared to 254 nm irradiation.^14^ Wastewater treatment plant effluents can contain nitrate levels up to 30 mg-N/L. In agricultural watersheds, surface water nitrate concentrations often range from 2 to over 10 mg/L. Stormwater runoff nitrate concentrations can also vary widely, influenced by factors such as land use and rainfall patterns.^83–85^ Under such conditions, Far-UVC treatment could harness this naturally occurring nitrate as a radical-generating constituent, accelerating virus disinfection and providing a chemical abatement strategy that could be very relevant for decentralized treatment solutions in rural, peri-urban, or resource-limited areas. However, this approach is not without caveats. While the generation of RNS such as NO_2_• and peroxynitrite (ONOO^–^) can contribute to viral inactivation and chemical degradation, these species may also participate in unwanted side reactions, including the formation of nitro-aromatic compounds (e.g., nitrophenols) and if a postchlorination step is used, can form disinfection byproducts (DBPs) like halonitromethanes.^22,86^ Additionally, if chlorine or chloramines are present in the water matrix before UV222 application, reactive chlorine species (RCS) can be produced, which may further react with RNS and DOM. This interplay can increase the risk of chlorinated and nitrogenous DBP formation via synergistic RNS–RCS pathways.^87^ Therefore, any deployment of nitrate-enhanced Far-UVC processes must be accompanied by an assessment of the potential formation of transformation products and related toxicity to ensure that the benefits of improved disinfection do not come at the expense of downstream water quality. The water disinfection application of KrCl* excimer lamps is influenced by the water’s ultraviolet transmittance (UVT) and is affected by the presence of organic matter, which can attenuate UV photons and reduce disinfection efficacy. This study investigated the unfiltered KrCl* excimer performance under controlled conditions with minimal background absorption and low nitrate concentrations. However, other scenarios, such as aquaculture systems where chlorine cannot be used, and nitrified wastewater treatment plant effluents, often exhibit higher levels of organic matter and nitrate. These constituents can significantly impact the generation of reactive oxygen species, weakening or even completely inhibiting the overall microbial inactivation process. Moreover, implementing 222 nm Far UVC in flow-through systems requires consideration of two key factors: (1) KrCl excimer lamps produce significantly lower radiant flux, typically a few hundred milliwatts compared to 1–1000 W from LP mercury lamps.^88^ They also exhibit low energy efficiency (around 2–12%), while LP lamps convert 30–40% of input power into UV output.^89,90^ (2) Effective deployment demands unique reactor design due to the low penetration of UV222 in complex water matrices that ensures proper mixing, optical clarity, and flow rates aligned with real-world infrastructure.^34^ These disparities, with the low radiant flux and level of technology maturity, could result in increased energy use and low lamp lifespan in scaled systems. Ultimately, while UV/nitrate treatment presents a promising avenue for water disinfection, water quality impacts and the scalability of systems to continuous flow operations are challenges.

## 4 Conclusions

This study demonstrates that 222 nm unfiltered KrCl* excimer irradiation can substantially enhance inactivation compared to 254 nm conventional LP lamp technology, particularly through combined mechanisms of direct photolysis and nitrate-induced advanced oxidation. Among the tested organisms, MS2 bacteriophage exhibited significant sensitivity to UV222 in the presence of nitrate, driven primarily by direct 222 nm effectiveness and hydroxyl radical activity, while the role of reactive nitrogen species was minor. In contrast, the disinfection of T1UV and *P. aeruginosa* was dominated by direct photolysis, with nitrate addition offering limited benefit. T1UV remained largely unaffected by radical attack, due to its complex capsid and tail structure. In contrast, MS2’s protein and RNA structure are more susceptible to oxidative damage. These results highlight the importance of organism-specific structural and biochemical characteristics in determining susceptibility to radical-mediated disinfection. While nitrate photolysis under 222 nm UV presents a promising pathway for enhancing disinfection in nitrate-rich waters, potential unintended byproduct formation necessitates further investigation under a diversity of water matrices to optimize application and safeguard water quality.

## Supplementary Material



## Notes

Description of MS2 and T1UV methods; MS2 inactivation under different TBA conditions; calculation of reaction rate of RNS with uric acid and MS2; experimental conditions for controls; inactivation rate constants of bacteriophage under difference conditions; photolysis pathway from nitrate to oxidants; nitrite formation; log reduction values under UV222; MS2 dark control experiments; rate calculation of RNS production; inactivation of MS2 under different conditions; model parameters for *P. aeruginosa* inactivation; values and reactions used in Kintecus kinetic model; Kintecus model results; Supplementary References (PDF)
