# Supplementary material for: Role of Nitrate-Driven Radical Formation in Microorganism Inactivation under 222 nm UV Irradiation
Source: ACS ES T Water. 2026 May 28;6(6):3555–67. doi: 10.1021/acsestwater.5c01533 (PMC13270515; doi:10.1021/acsestwater.5c01533)
Supplement: Supplementary file 1 [file ew5c01533_si_001.pdf]

Role of nitrate-driven radical formation in microorganism inactivation under 222 nm UV irradiation

Dana Pousty, Emma M. Payne, Karl G. Linden*

Department of Civil, Environmental, and Architectural Engineering, University of Colorado Boulder, 4001 Discovery Dr., Boulder, CO 80303, United States

Text S1. MS2 stock concentration of ~10^{11} PFU/ml, was prepared using methods adopted from previous studies, as described (Kundu et al., 2023; Pousty et al., 2025). Briefly, the host bacteria, transferred from a single colony, were incubated with constant stirring at 37 °C for 24 h. Then, 1.5 mL of the host was transferred to fresh 150 mL TSB with and incubated at 37 °C for ~6 hours until reaching a log growth phase. MS2 phage stock was added to the host and incubated for ~18 hours following centrifugation (10 min, 8000×G) and filtration through a 0.22-μm low protein binding filter (PES Stericup filter, Millipore) to remove cellular debris. The stock MS2 (~10^{12} PFU mL^{-1}) was kept at 4 °C.

T1UV stock concentration of ~10^{10} PFU/ml, was prepared using methods adopted from previous studies, as described. Briefly, the host bacteria (*E. coli CN-13*), transferred from a single colony, were incubated with constant stirring at 37 °C for 24 h. Then, 0.1 mL of the host was transferred to fresh 0.5 mL TSB with and incubated at 35 °C for 4-5 hours until reaching a log growth phase. T1UV phage stock was added to the host and incubated for ~18 hours following centrifugation (10 min, 8000×G) and filtration through a 0.22-μm low protein binding filter (PES Stericup filter, Millipore) to remove cellular debris. The stock T1UV (~10^{12} PFU mL^{-1}) was kept at 4 °C.

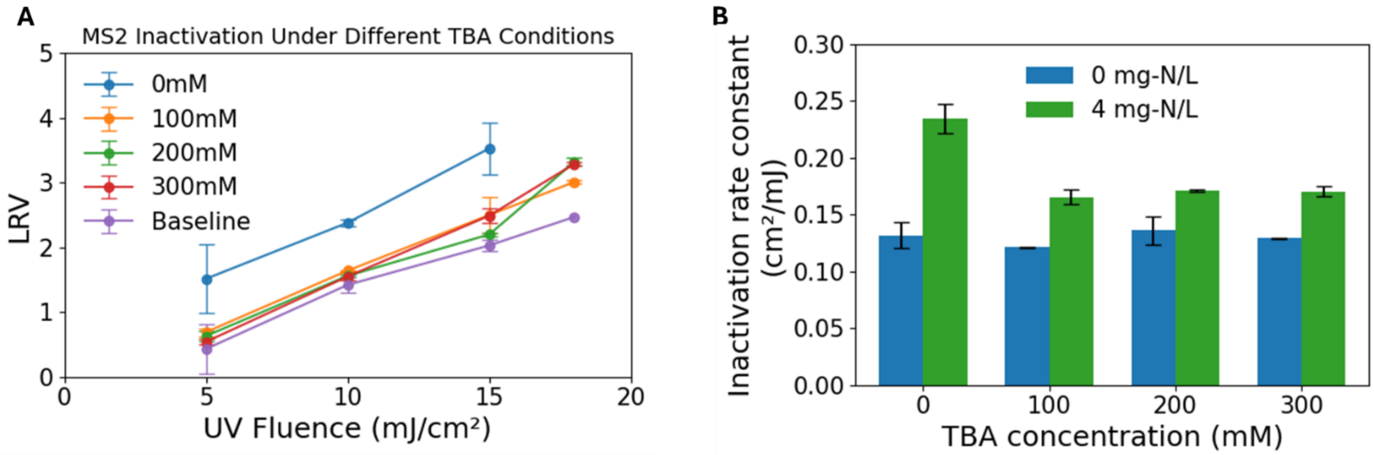

Figure S1. (A) Inactivation of MS2 bacteriophage with 4 mg-N/L of nitrate concentration under UV222 at varying TBA concentrations: 0, 100, 200 and 300 mM (legend refers to TBA concentration), Baseline represents TBA at 0mM and 0 mg-N/L. (B) MS2 inactivation rate constant at different initial nitrate concentrations and different TBA concentration (legend refers to nitrate concentration). Log reduction value (LRV) was calculated using Eq. (1), inactivation rate constant was calculated using Eq. (2). Error bars represent one standard deviation around the mean (n=3).

Text S2. Calculation of reaction rate of NO_2• with uric acid and MS2

$$NO_2 \bullet\ + \mathrm{UA}\ \rightarrow \mathrm{Product}$$

$[NO_2 \bullet] =\ 2.0 \times 10^{-7} M$ Based on our Kintecus model, Fig S5

$[UA] =$ can be ignored under the pseudo first order assumption

$k_1 = 1.8 \times 10^{7}\ M^{-1} S^{-1}$ (Pham et al., 2021)

$$r_1 =\ k_1 [UA][NO_2 \bullet] = (1.8 \times 10^{7}\ M^{-1} S^{-1}) \times (1.91 \times 10^{-7} M) = 3.44\ s^{-1}$$

$$NO_2 \bullet\ + \mathrm{MS2}\ \rightarrow \mathrm{Product}$$

$[MS2] =$ can be ignored under the pseudo first order assumption

$k_2 [NO_2 \bullet] = (1.17 \times 10^{5}\ M^{-1} S^{-1})$ = k_{app}(RNS– organism) ($M^{-1} \cdot s^{-1}$) determined in section 3.4

$$r_2 =\ k_2 [MS2][NO_2 \bullet] = (1.17 \times 10^{5}\ M^{-1} S^{-1}) \times (2.0 \times 10^{-8} M) = 0.00234\ s^{-1}$$

$r_1 \gg\ r_2$ - UA is the dominant sink for NO_2• under the experimental conditions

Table S1. Experimental conditions for all controls without UV irradiation.

**Organism**	**Nitrate concentration (mg-N/L)**	**TBA concentration (mM)**	**Uric acid concentration (µM)**
**MS2**	Direct (0)	0,100,200,300	0,400,800
	4	0,100,200,300	0,400,800
	8	-	-
**TIUV**	Direct (0),4,8	0,100,200,300	-
P. aeruginosa	Direct (0),4,8	-	-

Table S2. Inactivation rate constant of MS2 and T1UV bacteriophage under different UV sources and nitrate concentrations.

Organism	UV source	Nitrate concentration (mg-N/L)	*k (direct) and k(NO_3) (cm²/mJ)*	*k (NO_3+TBA) (cm²/mJ)*	*k (NO_3+TBA+UC) (cm²/mJ)*
MS2	UV 254	0	0.067±0.006	-	-
		4	0.037±0.001	-	-
		8	0.041±0.002	-	-
	UV222	0	0.134±0.013	0.129	0.136
		4	0.241±0.011	0.170	0.154
		8	0.215±0.006	0.170	0.154
T1UV		0	0.341±0.014	0.341	-
		4	0.421±0.008	0.339	-
		8	0.369±0.003	0.339	-

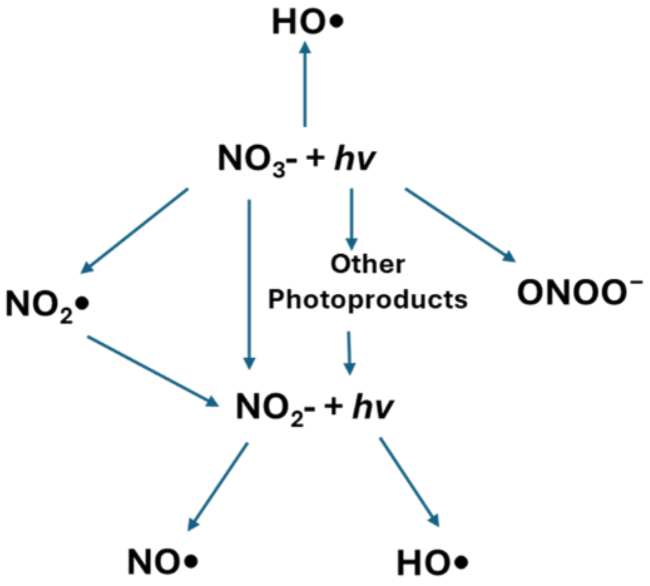

Fig S2. Simplified photolysis pathway from nitrate to ROS and RNS (adapted from (Keen et al., 2012)

Table S3. Simplified photolysis pathway from nitrate to ROS and RNS

Reaction	Reference
$\mathbf{NO_3^- + hv \rightarrow NO_2^\bullet + \bullet OH}$	(Yin et al., 2024b)
$\mathbf{NO_3^- + hv \rightarrow NO_2^- + O}$ (3P)	(Yin et al., 2024b)
$\mathbf{NO_3^- + hv \rightarrow ONOO^-}$	(Yin et al., 2024b)
$\mathbf{OONOO^- \rightarrow NO_2^\bullet + O_2^{\bullet -}}$	[4]
$\mathbf{OONOO^- \rightarrow NO_2^\bullet + OH^-}$	[5]
$\mathbf{OONOO^- \rightarrow NO_2^- + O_2}$	[4]
$\mathbf{NO_2^\bullet + O^{\bullet -} \rightarrow \bullet OH}$	(Keen et al., 2012)

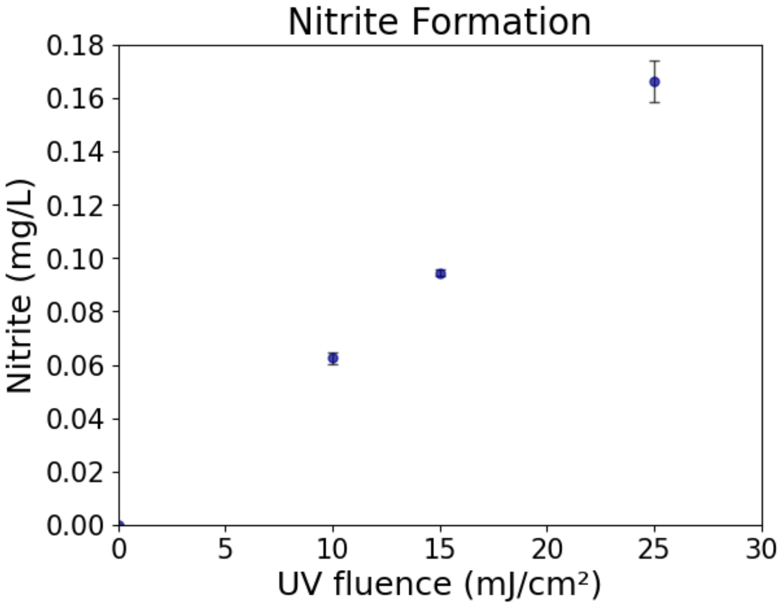

Fig. S3. Nitrite concentration at different UV fluences with 4 mg-N/L nitrate and MS2 bacteriophage. Error bars represent one standard deviation of the means (n=2).

Table S4. The log reduction value and exposure time of different microorganisms at UV fluence of 10 mJ/cm^2 under UV222

Microorganism	Nitrate concentration (mg-N/L)	LRV ± STDV	Exposure time (Sec)
**MS2**	Direct (0)	1.41±0.12	19
	4	2.38±0.06	20
	8	2.27±0.18	20
**T1UV**	Direct (0)	3.42±0.31	18
	4	3.98±0.48	18
	8	3.68±0.51	18
P. aeruginosa	Direct (0)	4.78±0.06	12
	4	4.93±0.03	14
	8	4.70±0.45	14

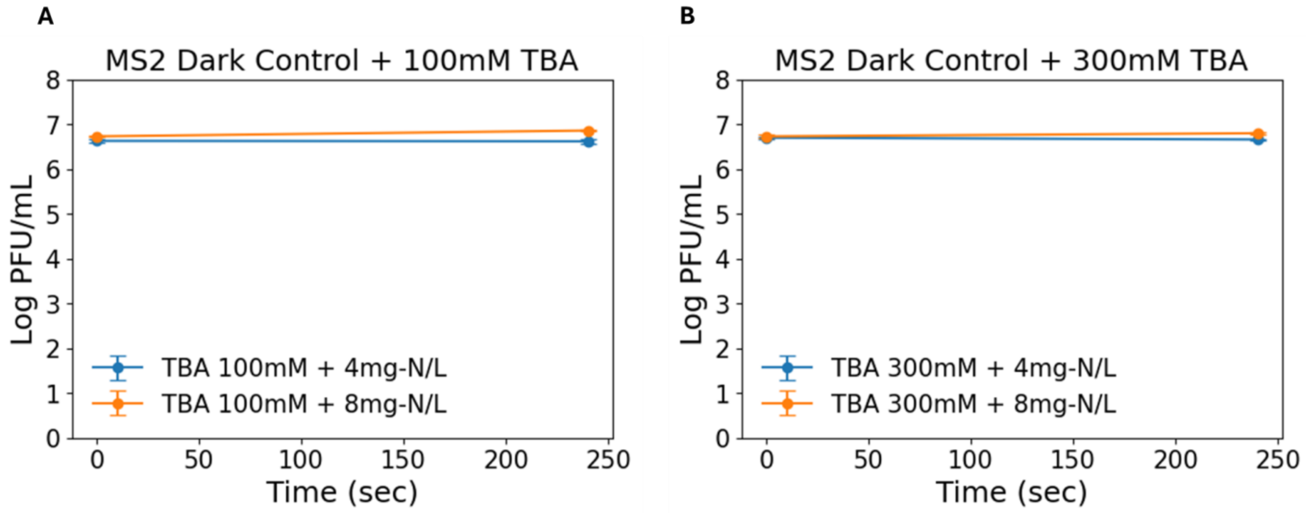

Fig. S4. Dark control experiments for MS2 with (A) TBA concentration at 100mM (B) TBA concentration at 300mM, Error bars represent one standard deviation of the means (n=3).

Text S3. Rate calculation of $NO_2\bullet$ production via $\bullet OH$ + nitrite

$$NO_2^- + \bullet\,\mathrm{OH} \rightarrow NO_2\,\bullet$$

$$[\bullet\,\mathrm{OH}\,] = 1.59 \times 10^{-12} M \text{ Based on our Kintecus model, Fig S5}$$

$$[NO_2^-\,] = \text{ measured 0.0945 mg-N/L at 15 mJ/cm}^2 = 6.75 \times 10^{-6} M$$

$$k_1[\bullet\,\mathrm{OH}][NO_2^-] = (1.1 \times 10^9\, M^{-1} S^{-1}) \text{ at 25 °C, pH =7 } ^6$$

$$r_1 = k_1[\bullet\,\mathrm{OH}\,][NO_2^-] = (1.1 \times 10^9\, M^{-1} S^{-1})\,(1.59 \times 10^{-12} M\,) \times (6.75 \times 10^{-6} M)$$
$$= 1.18 \times 10^{-8}\, s^{-1}$$

$$NO_3^- \rightarrow NO_2^\bullet + O^{\bullet -}$$

$$r_2 = 2.29 \times 10^{-7}\, s^{-1} \text{ According to Kintecus model, Table S5}$$

$r_2 \gg r_1$ - direct nitrate photolysis produces $NO_2\bullet$ an order of magnitude faster than the $\bullet$ OH + nitrite pathway

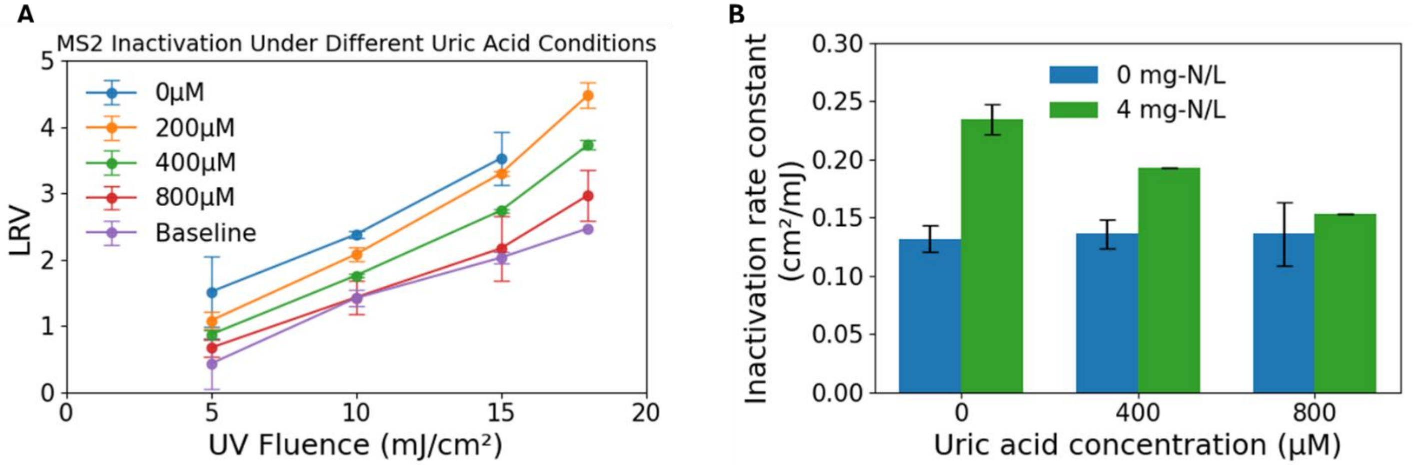

Figure S5. (A) Inactivation of MS2 bacteriophage with 4 mg-N/L of nitrate and 200 mM TBA under UV222 at Uric acid concentrations of 0, 400, and 800 µM (legend refers to Uric acid concentration), Baseline represents uric acid 0µM, TBA at 0 mM, and nitrate at 0 mg-N/L. (B) MS2 inactivation rate constant at different initial nitrate concentrations and different uric acid concentrations (legend refers to nitrate concentration). Conditions: 200 mM TBA concentration for quenching OH radical activity. Log reduction value (LRV) was calculated using Eq. (1), inactivation rate constant was calculated using Eq. (2). Error bars represent one standard deviation of the means (n=3)

Table S5. Weibull model parameters for *P. aeruginosa* inactivation under UV222 nm at different nitrate concentrations

**Nitrate concentration (mg-N/L)**	**δ (scale)**	**p (shape)**	**R^2**	**RMSE**
**Direct (0)**	0.813	0.592	0.959	0.386
**4**	0.672	0.587	0.975	0.322
**8**	0.825	0.581	0.930	0.495

Table S6. Values used in Kintecus kinetic model when evaluating initial nitrate concentration of 4 mg-N/L.

Nitrate concentration - 0.00028 [M] – 4 mg-N/L			
Photolysis of Nitrate to	$NO_2^{\bullet} + O^{\bullet -}$	$NO_2^{-} + O$	$ONOO^{-}$
Quantum Yield for rxn (mol ein^{-1})	0.113	0.054	0.27
Path Length (cm)	0.95	0.95	0.95
Absorbance (cm^{-1})	0.5220	0.5220	0.5220
Absorbance (A) (unitless)	0.4959	0.4959	0.4959
Molar Extinction Coeff (M^{-1} cm^{-1})	2747	2747	2747
Incident Irradiance (I_0) (mW cm^{-2})	0.623	0.623	0.623
Photon Energy (U, 222nm) (J Ein^{-1})	5.38E+05	5.38E+05	5.38E+05
Photon Flux / unit area (ein s^{-1} cm^{-2})	1.16E-09	1.16E-09	1.16E-09
Petri Dish Area (cm^2)	8.55	8.55	8.55
Photon Flux, overall (Ein s^{-1})	9.90E-09	9.90E-09	9.90E-09
Volume solution (L)	5.00E-03	5.00E-03	5.00E-03
f, solution (fraction of light absorbed by solution)	0.681	0.681	0.681
Photolysis Rate (mol s^{-1})	2.29E-07	1.09E-07	5.47E-07
1st Order Photolysis Rate: k,NO_3^- (s^{-1})	8.02E-04	3.83E-04	1.92E-03

Table S7. Values used in Kintecus kinetic model when evaluating initial nitrate concentration of 8 mg-N/L.

Nitrate concentration - 0.00057 [M] – 8 mg-N/L			
Photolysis of Nitrate to	$NO_2^{\bullet} + O^{\bullet-}$	$NO_2^- + O$	$ONOO^-$
Quantum Yield for rxn (mol ein^{-1})	0.113	0.054	0.27
Path Length (cm)	0.95	0.95	0.95
Absorbance (cm^{-1})	1.2700	1.2700	1.2700
Absorbance (A) (unitless)	1.2065	1.2065	1.2065
Molar Extinction Coeff (M^{-1} cm^{-1})	2747	2747	2747
Incident Irradiance (I_0) (mW cm^{-2})	0.623	0.623	0.623
Photon Energy (U, 222nm) (J Ein^{-1})	5.38E+05	5.38E+05	5.38E+05
Photon Flux / unit area (ein s^{-1} cm^{-2})	1.16E-09	1.16E-09	1.16E-09
Petri Dish Area (cm^2)	8.55	8.55	8.55
Photon Flux, overall (Ein s^{-1})	9.90E-09	9.90E-09	9.90E-09
Volume solution (L)	5.00E-03	5.00E-03	5.00E-03
f, solution (fraction of light absorbed by solution)	0.938	0.938	0.938
Photolysis Rate (mol s^{-1})	2.59E-07	1.24E-07	6.20E-07
1st Order Photolysis Rate: k,NO_3^- (s^{-1})	4.54E-04	2.17E-04	1.08E-03

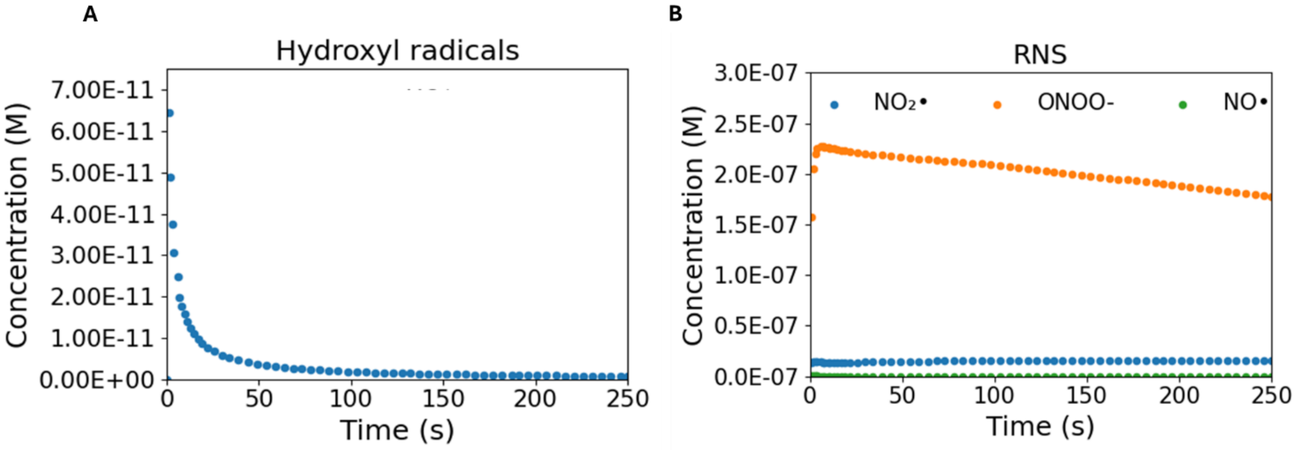

Figure S6. Kintecus kinetic model results for Nitrate concentration - 0.00028 [M] – 4 mg-N/L

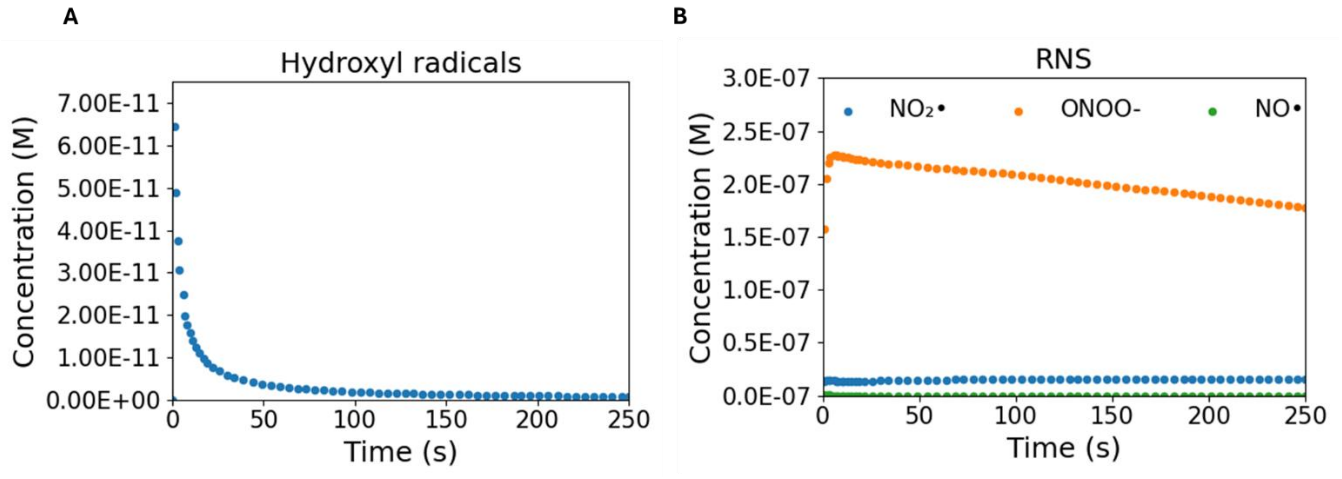

Figure S7. Kintecus kinetic model results for Nitrate concentration of 0.00057 [M] – 8 mg-N/L

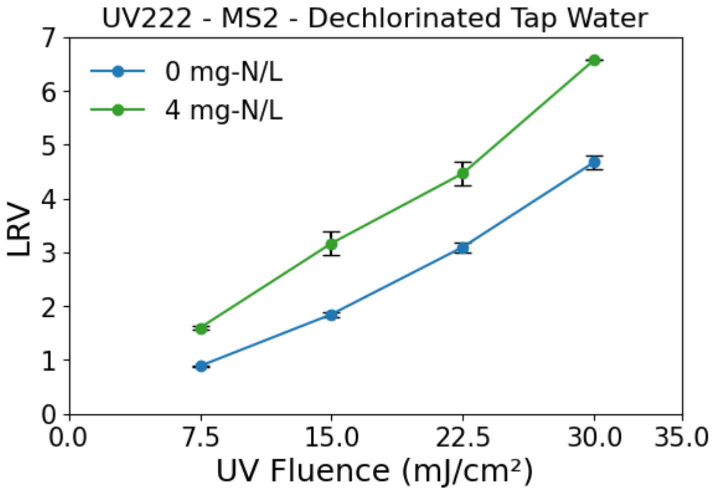

Figure S8. Inactivation of MS2 bacteriophage under UV222 (at varying nitrate concentrations: 0, 4 mg-N/L (legend refers to nitrate concentration) in dechlorinated tap water. Log reduction value (LRV) was calculated using Eq. (1).Error bars represent one standard deviation of the means (n = 3).

Table S8. Reactions included in Kintecus kinetic model, adapted from Yin, et al (2024)[7]

No.	Rate Constant	Reaction	Reference
Photolysis reactions			
**1**	1.23 x10^{-3}	$NO_3^- + hv \rightarrow NO_2^{\bullet} + O^{\bullet -}$	
**2**	5.86 x10^{-4}	$NO_3^- + hv \rightarrow NO_2^- + O$	
**3**	2.93 x10^{-3}	$NO_3^- + hv \rightarrow ONOO^-$	
RNS reactions			
**8**	1.00	$OONOO^- \rightarrow NO_2^{\bullet} + O_2^{\bullet -}$	4
**9**	1.00	$OONOO^- \rightarrow NO_2^{\bullet} + OH^-$	5
**10**	1.40	$OONOO^- \rightarrow NO_2^- + O_2$	4
**11**	2.60 x10^{-2}	$ONOOHO \rightarrow HO_2^{\bullet} + NO_2^{\bullet}$	8
**12**	7.00 x10^{-4}	$ONOOHO \rightarrow H^+ + NO_2^- + O_2$	8
**13**	1.25 x10^{4}	$ONOOH \rightarrow ONOO^- + H^+$	9
**14**	1.40	$ONOOH \rightarrow NO_3^- + H^+$	10
**15**	3.50 x10^{-1}	$ONOOH \rightarrow NO_2^{\bullet} + {}^{\bullet}OH$	9
**16**	1.71 x10^{-4}	$ONOOH \rightarrow HO_2^{\bullet} + NO^{\bullet}$	9
**17**	1.10 x10^{-1}	$ONOOH + H_2O \rightarrow HNO_2 + H_2O_2$	4
**18**	4.30	$ONOOH \rightarrow NO_3^- + H^+$	11
**19**	8.00 x10^{-6}	$ONOO^- \rightarrow NO_3^-$	4
**20**	1.00 x10^{-6}	$ONOO^- \rightarrow NO_2^{\bullet} + O_2^{\bullet -}$	9
**21**	2.00 x10^{-2}	$ONOO^- \rightarrow NO^{\bullet} + O_2^{\bullet -}$	9
**22**	4.80 x10^{9}	$ONOO^- + {}^{\bullet}OH \rightarrow NO^{\bullet} + O_2 + OH^-$	9
**23**	5.00 x10^{10}	$ONOO^- + H^+ \rightarrow ONOOH$	9
**24**	2.90 x10^{4}	$ONOO^- + CO_3^{\bullet -} \rightarrow NO^{\bullet} + O_2 + CO_3^{2-}$	9
**25**	5.80 x10^{4}	$ONOO^- + CO_2 \rightarrow ONOOCO_2^-$	11
**26**	6.70 x10^{5}	$ONOO^- + CO_2 \rightarrow NO_3^- + CO_2$	11
**27**	2.90 x10^{4}	$ONOO^- + CO_2 \rightarrow NO_2^{\bullet} + CO_3^{\bullet -}$	10
**28**	4.50 x10^{9}	$O_2^{\bullet -} + NO_2^{\bullet} \rightarrow OONOO^-$	11
**29**	4.00 x10^{4}	$NO_3^- + NO^{\bullet} \rightarrow NO_2^{\bullet} + NO_2^-$	9
**30**	4.00 x10^{5}	$NO_3^- + {}^{\bullet}OH \rightarrow NO_3^{\bullet} + OH^-$	5
**31**	9.70 x10^{9}	$NO_3^- + e^- \rightarrow NO_3^{2-}$	9
**32**	8.00 x10^{-4}	$NO_2^{\bullet} \rightarrow NO_2(g)$	8
**33**	1.10 x10^{-2}	$NO_2^{\bullet} \rightarrow NO^{\bullet} + O$	8
**34**	3.50 x10^{9}	$NO_2^{\bullet} + O^{\bullet -} \rightarrow ONOO^-$	9
**35**	4.50 x10^{8}	$NO_2^{\bullet} + NO_2^{\bullet} \rightarrow N_2O_4$	12
**36**	1.10 x10^{9}	$NO_2^{\bullet} + NO^{\bullet} \rightarrow N_2O_3$	9
**37**	4.50 x10^{9}	$NO_2^{\bullet} + HO_2^{\bullet} \rightarrow ONOOHO$	8
**38**	4.50 x10^{9}	$NO_2^{\bullet} + {}^{\bullet}OH \rightarrow ONOOH$	11
**39**	1.00 x10^{9}	$NO_2^{\bullet} + CO_3^{\bullet -} \rightarrow NO_3^- + CO_2$	9
**40**	3.00 x10^{-6}	$NO_2^- \rightarrow NO_2^{\bullet} + e^-$	9
**41**	4.30 x10^{-5}	$NO_2^- \rightarrow NO^{\bullet} + O^{\bullet -}$	9

**42**	5.00 x10^{6}	$NO_2^- + O_2^{\bullet-} \rightarrow X$	13
**43**	1.00 x10^{10}	$NO_2^- + {}^{\bullet}OH \rightarrow NO_2^{\bullet} + OH^-$	9
**44**	5.00 x10^{10}	$NO_2^- + H^+ \rightarrow HNO_2$	9
**45**	6.60 x10^{5}	$NO_2^- + CO_3^{\bullet-} \rightarrow NO_2^{\bullet} + CO_3^{2-}$	12
**46**	6.70 x10^{9}	$NO^{\bullet} + O_2^{\bullet-} \rightarrow ONOO^-$	9
**47**	2.10 x10^{6}	$2NO^{\bullet} + O_2 \rightarrow 2NO_2^{\bullet}$	9
**48**	3.20 x10^{9}	$NO^{\bullet} + HO_2^{\bullet} \rightarrow ONOOH$	11
**49**	1.70 x10^{10}	$NO^{\bullet} + {}^{\bullet}OH \rightarrow NO_2^- + H^+$	9
**50**	1.20 x10^{4}	$NO^{\bullet} + {}^{\bullet}OH \rightarrow HNO_2$	9
**51**	3.50 x10^{9}	$NO^{\bullet} + CO_3^{\bullet-} \rightarrow NO_2^- + CO_2$	9
**52**	6.90 x10^{3}	$N_2O_4 \rightarrow 2NO_2^{\bullet}$	9
**53**	1.00 x10^{3}	$N_2O_4 + H_2O \rightarrow NO_2^- + NO_3^- + 2H^+$	14
**54**	2.10 x10^{6}	$N_2O_3 \rightarrow NO^{\bullet} + NO_2^{\bullet}$	9
**55**	3.10 x10^{8}	$N_2O_3 + ONOO^- \rightarrow NO_2^{\bullet} + NO_2^-$	9
**56**	1.90 x10^{6}	$N_2O_3 + HCO_3^- \rightarrow NO_2^- + HCO_3NO$	9
**57**	2.88 x10^{2}	$N_2O_3 + H_2O \rightarrow 2NO_2^- + 2H^+$	9
**58**	1.00 x10^{-3}	$HNO_2 \rightarrow NO^{\bullet} + {}^{\bullet}OH$	9
**59**	2.60 x10^{9}	$HO^{\bullet} + HNO_2 \rightarrow H_2O + NO_2^{\bullet}$	15
**60**	9.60 x10^{3}	$HNO_2 + H_2O_2 \rightarrow ONOOH + H_2O$	9
**61**	5.50 x10^{4}	$NO_3^{2-} + H_2O \rightarrow NO_2^{\bullet} + 2OH^-$	9
ROS reactions			
**62**	1.94 x10^{3}	$O_3^{\bullet-} \rightarrow O_2 + O^{\bullet-}$	16
**63**	9.00 x10^{8}	$O_3^{\bullet-} + O_3^{\bullet-} \rightarrow X$	16
**64**	7.00 x10^{8}	$O_3^{\bullet-} + O^{\bullet-} \rightarrow O_4^{2-}$	16
**65**	7.00 x10^{8}	$O_3^{\bullet-} + O^{\bullet-} \rightarrow O_2^{\bullet-} + O_2^{\bullet-}$	16
**66**	9.00 x10^{10}	$O_3^{\bullet-} + H^+ \rightarrow O_2 + {}^{\bullet}OH$	16
**67**	6.00 x10^{8}	$O_2^{\bullet-} + O^{\bullet-} + H_2O \rightarrow O_2 + 2OH^-$	16
**68**	1.30 x10^{-1}	$O_2^{\bullet-} + H_2O_2 \rightarrow O_2 + {}^{\bullet}OH$	16
**69**	2.00 x10^{10}	$O_2^{\bullet-} + H^+ \rightarrow HO_2^{\bullet}$	16
**70**	3.50 x10^{9}	$O_2 + O^{\bullet-} \rightarrow O_3^{\bullet-}$	16
**71**	4.70 x10^{9}	$O^{\bullet-} + O^{\bullet-} \rightarrow O_2^{2-}$	16
**72**	4.00 x10^{8}	$O^{\bullet-} + H_2O_2 \rightarrow O_2^{\bullet-} + H_2O$	16
**73**	1.70 x10^{6}	$O^{\bullet-} + H_2O \rightarrow HO^{\bullet} + OH^-$	17
**74**	1.00 x10^{11}	$O^{\bullet-} + H^+ \rightarrow HO^{\bullet}$	16
**75**	3.20 x10^{5}	$HO_2^{\bullet} \rightarrow O_2^{\bullet-} + H^+$	16
**76**	9.70 x10^{7}	$HO_2^{\bullet} + O_2^{\bullet-} \rightarrow HO_2^- + O_2$	16
**77**	8.30 x10^{9}	$HO_2^{\bullet} + HO_2^{\bullet} \rightarrow H_2O_2 + O_2$	16
**78**	3.00	$HO_2^{\bullet} + H_2O_2 \rightarrow O_2 + {}^{\bullet}OH + H_2O$	16
**79**	2.00	$HO_2^- + O_2^{\bullet-} \rightarrow H_2O_2 + O_2$	16
**80**	4.00 x10^{8}	$HO_2^- + O^{\bullet-} \rightarrow HO^{\bullet} + O_2^{\bullet-}$	16
**81**	5.00 x10^{8}	$HO_2^- + O^{\bullet-} + H_2O \rightarrow HO_2^{\bullet} + 2OH^-$	16
**82**	3.00 x10^{7}	$HO_2^- + CO_3^{\bullet-} \rightarrow CO_3^{2-} + HO_2^{\bullet}$	16

**83**	1.30 x10^{-1}	$HO^{\bullet} \rightarrow O^{\bullet-} + H^{+}$	16
**84**	1.25 x10^{10}	$HO^{\bullet} + OH^{-} \rightarrow O^{\bullet-} + H_2O$	16
**85**	1.20 x10^{10}	$HO^{\bullet} + O_2^{\bullet-} \rightarrow O_2 + OH^{-}$	16
**86**	1.10 x10^{5}	$HO^{\bullet} + O_2 \rightarrow O_3^{\bullet-} + H^{+}$	16
**87**	2.00 x10^{10}	$HO^{\bullet} + O^{\bullet-} \rightarrow HO_2^{-}$	16
**88**	7.10 x10^{9}	$HO^{\bullet} + HO_2^{\bullet} \rightarrow O_2 + H_2O$	16
**89**	7.50 x10^{9}	$HO^{\bullet} + HO_2^{-} \rightarrow OH^{-} + HO_2^{\bullet}$	16
**90**	7.10 x10^{9}	$HO^{\bullet} + HO_2^{-} \rightarrow H_2O + O_2^{\bullet-}$	16
**91**	5.50 x10^{9}	$HO^{\bullet} + HO^{\bullet} \rightarrow H_2O_2$	16
**92**	2.70 x10^{7}	$HO^{\bullet} + H_2O_2 \rightarrow H_2O + HO_2^{\bullet}$	16
**93**	2.70 x10^{7}	$H_2O_2 + HO^{\bullet} \rightarrow H_2O + H^{+} + O_2^{\bullet-}$	5
**94**	1.90 x10^{10}	$e^{-} + O_2 \rightarrow O_2^{\bullet-}$	15
Carbonate reactions			
**95**	1.00 x10^{6}	$HO^{\bullet} + H_2CO_3 \rightarrow CO_3^{\bullet-} + H_2O + H^{+}$	4
**96**	8.50 x10^{6}	$HO^{\bullet} + HCO_3^{-} \rightarrow CO_3^{\bullet-} + H_2O$	18
**97**	3.90 x10^{8}	$HO^{\bullet} + CO_3^{2-} \rightarrow CO_3^{\bullet-} + OH^{-}$	18
**98**	3.00 x10^{9}	$HO^{\bullet} + CO_3^{\bullet-} \rightarrow X$	4
**99**	4.30 x10^{5}	$H_2O_2 + CO_3^{\bullet-} \rightarrow HCO_3^{-} + HO_2^{\bullet}$	19
**100**	6.00 x10^{8}	$CO_3^{\bullet-} + O_2^{\bullet-} \rightarrow CO_3^{2-} + O_2$	4
**101**	3.00 x10^{7}	$CO_3^{\bullet-} + HO_2^{-} \rightarrow CO_3^{2-} + HO_2^{\bullet}$	4
**102**	8.00 x10^{5}	$CO_3^{\bullet-} + H_2O_2 \rightarrow HO_2^{\bullet} + HCO_3^{-}$	20
**103**	8.00 x10^{5}	$CO_3^{\bullet-} + H_2O_2 \rightarrow HCO_3^{-} + O_2^{\bullet-} + H^{+}$	21
**104**	5.00 x10^{10}	$H^{+} + HCO_3^{-} \rightarrow H_2CO_3$	22
**105**	2.50 x10^{4}	$H_2CO_3 \rightarrow HCO_3^{-} + H^{+}$	22
**106**	3.60 x10^{2}	$H_2CO_3 + H_2O \rightarrow H^{+} + HCO_3^{-}$	22
**107**	6.75 x10^{-4}	$CO_2 + H_2O \rightarrow H_2CO_3$	22
**108**	2.00 x10^{-4}	$HCO_3^{-} \rightarrow CO_2 + OH^{-}$	23
**109**	8.30 x10^{3}	$CO_2 + OH^{-} \rightarrow HCO_3^{-}$	22
**110**	4.20 x10^{-2}	$HCO_3^{-} + H_2O \rightarrow CO_3^{2-} + H^{+}$	22
**111**	5.00 x10^{10}	$CO_3^{2-} + H^{+} \rightarrow HCO_3^{-} + H_2O$	22
**112**	6.00 x10^{9}	$HCO_3^{-} + OH^{-} \rightarrow CO_3^{2-} + H_2O$	22
**113**	2.30 x10^{4}	$CO_3^{2-} + H_2O \rightarrow HCO_3^{-} + OH^{-}$	22
**114**	4.25 x10^{6}	$CO_3^{\bullet-} + CO_3^{\bullet-} \rightarrow CO_2 + CO_4^{2-}$	20
**115**	2.00 x10^{8}	$CO_3^{\bullet-} + CO_4^{2-} \rightarrow CO_3^{2-} + CO_4^{\bullet-}$	20
**116**	1.00 x10^{9}	$CO_3^{\bullet-} + CO_4^{\bullet-} \rightarrow C_2O_7^{2-}$	20
Equilibrium reactions			
**117**	2.51 x10^{7}	$HNO_2 \rightarrow NO_2^{-} + H^{+}$	9
**118**	1.20 x10^{-1}	$H_2O_2 \rightarrow HO_2^{-} + H^{+}$	16
**119**	1.00 x10^{-3}	$H_2O \rightarrow H^{+} + OH^{-}$	16
**120**	1.00 x10^{11}	$H^{+} + OH^{-} \rightarrow H_2O$	16
**121**	5.00 x10^{10}	$H^{+} + HO_2^{-} \rightarrow H_2O_2$	16
Phosphate reactions			

**122**	3.97 x10^{8}	$H_3PO_4 \rightarrow H_2PO_4{}^- + \mathrm{H}^+$	16
**123**	5.00 x10^{10}	$HPO_4{}^{2-} + \mathrm{H}^+ \rightarrow H_2PO_4{}^-$	16
**124**	5.00 x10^{10}	$H_2PO_4{}^- + \mathrm{H}^+ \rightarrow H_3PO_4$	16
**125**	3.15 x10^{3}	$H_2PO_4{}^- \rightarrow HPO_4{}^{2-} + \mathrm{H}^+$	16
**126**	5.00 x10^{10}	$PO_4{}^{3-} + \mathrm{H}^+ \rightarrow HPO_4{}^{2-}$	16
**127**	2.50 x10^{-2}	$HPO_4{}^{2-} \rightarrow PO_4{}^{3-} + \mathrm{H}^+$	16
**128**	1.50 x10^{5}	$HPO_4{}^{2-} + \mathrm{HO}^\bullet \rightarrow HPO_4{}^{\bullet-} + \mathrm{OH}^-$	16
**129**	3.00 x10^{8}	$HPO_4{}^{\bullet-} + HPO_4{}^{\bullet-} \rightarrow P_2O_8 + 2\mathrm{H}^+$	16
Chloride reactions			
**130**	5.00 x10^{10}	$\mathrm{H^+ + OCl^- \rightarrow HOCl}$	24
**131**	1.60 x10^{3}	$\mathrm{HOCl \rightarrow H^+ + OCl^-}$	24
**132**	5.00 x10^{10}	$\mathrm{H^+ + Cl^- \rightarrow HCl}$	24
**133**	8.60 x10^{16}	$\mathrm{HCl \rightarrow H^+ + Cl^-}$	24
**135**	4.30 x10^{9}	$\mathrm{Cl^- + HO^\bullet \rightarrow ClOH^{\bullet-}}$	24
**136**	6.10 x10^{9}	$\mathrm{ClOH^{\bullet-} \rightarrow Cl^- + HO^\bullet}$	24
**137**	2.10 x10^{10}	$\mathrm{ClOH^{\bullet-} + H^+ \rightarrow Cl^\bullet + H_2O}$	24
**138**	1.00 x10^{4}	$\mathrm{ClOH^{\bullet-} + Cl^- \rightarrow Cl_2^{\bullet-} + OH^-}$	24
**139**	2.50 x10^{5}	$\mathrm{Cl^\bullet + H_2O \rightarrow ClOH^{\bullet-} + H^+}$	24
**140**	1.80 x10^{10}	$\mathrm{Cl^\bullet + OH^- \rightarrow ClOH^{\bullet-}}$	24
**141**	2.00 x10^{9}	$\mathrm{Cl^\bullet + H_2O_2 \rightarrow HO_2^\bullet + Cl^- + H^+}$	24
**142**	8.50 x10^{9}	$\mathrm{Cl^\bullet + Cl^- \rightarrow Cl_2^{\bullet-}}$	24
**143**	8.80 x10^{7}	$\mathrm{Cl^\bullet + Cl^\bullet \rightarrow Cl_2}$	24
**144**	3.00 x10^{9}	$\mathrm{Cl^\bullet + HOCl \rightarrow ClO^\bullet + H^+ + Cl^-}$	24
**145**	8.30 x10^{9}	$\mathrm{Cl^\bullet + OCl^- \rightarrow ClO^\bullet + Cl^-}$	24
**146**	6.00 x10^{4}	$\mathrm{Cl_2^{\bullet-} \rightarrow Cl^\bullet + Cl^-}$	24
**147**	1.00 x10^{9}	$\mathrm{Cl_2^\bullet + OH^\bullet \rightarrow HOCl + Cl^-}$	24
**148**	8.30 x10^{8}	$\mathrm{Cl_2^{\bullet-} + Cl_2^{\bullet-} \rightarrow Cl_2 + 2Cl^-}$	24
**149**	2.10 x10^{9}	$\mathrm{Cl_2^{\bullet-} + Cl^\bullet \rightarrow Cl_2 + Cl^-}$	24
**150**	1.40 x10^{5}	$\mathrm{Cl_2^{\bullet-} + H_2O_2 \rightarrow HO_2^\bullet + 2Cl^- + H^+}$	24
**151**	3.00 x10^{9}	$\mathrm{Cl_2^{\bullet-} + HO_2^\bullet \rightarrow} O_2 \mathrm{+ 2Cl^- + H^+}$	24
**152**	1.00 x10^{9}	$\mathrm{Cl_2^\bullet + O_2^{\bullet-} \rightarrow O_2 + 2Cl^-}$	24
**153**	2.34 x10^{1}	$\mathrm{Cl_2^{\bullet-} + H_2O \rightarrow Cl^- + HClOH}$	24
**154**	4.50 x10^{7}	$\mathrm{Cl_2^{\bullet-} + OH^- \rightarrow Cl^- + ClOH^{\bullet-}}$	24
**155**	5.40 x10^{8}	$\mathrm{Cl_2^{\bullet-} + OCl^- \rightarrow ClO^\bullet + 2Cl^-}$	24
**156**	1.00 x10^{8}	$\mathrm{HClOH \rightarrow ClOH^{\bullet-} + H^+}$	24
**157**	1.00 x10^{2}	$\mathrm{HClOH \rightarrow Cl^\bullet + H_2O}$	24
**158**	5.00 x10^{9}	$HClOH + Cl^- \rightarrow Cl_2^{\bullet-} + H_2O$	24
**159**	2.00 x10^{4}	$Cl_2 + Cl^- \rightarrow Cl_2^-$	24
**160**	1.10 x10^{5}	$Cl_3^- \rightarrow Cl_2 + Cl^-$	24
**161**	1.00 x10^{9}	$Cl_3^- + HO_2^\bullet \rightarrow Cl_2^{\bullet-} + HCl + O_2$	24
**162**	3.80 x10^{9}	$Cl_3^- + O_2^\bullet \rightarrow Cl_2^{\bullet-} + Cl^- + O_2$	24
**163**	2.70 x10^{1}	$Cl_2 + H_2O \rightarrow Cl^- + HOCl + H^+$	24

**164**	1.82×10^{1}	$Cl^- + HOCl + H^+ \rightarrow Cl_2 + H_2O$	[24]
**165**	1.30×10^{4}	$Cl_2 + H_2O_2 \rightarrow O_2 + 2HCl$	[24]
**166**	1.00×10^{9}	$Cl_2 + O_2^{\bullet-} \rightarrow O_2 + Cl_2^{\bullet-}$	[24]
**167**	1.00×10^{9}	$Cl_2 + HO_2^{\bullet} \rightarrow H^+ + O_2 + Cl_2^{\bullet-}$	[24]
**168**	1.00×10^{4}	$HOCl + H_2O_2 \rightarrow HCl + H_2O + O_2$	[24]
**169**	1.70×10^{5}	$OCl^- + H_2O_2 \rightarrow Cl^- + H_2O + O_2$	[24]
**170**	2.00×10^{9}	$HOCl + HO^\bullet \rightarrow ClO^\bullet + H_2O$	[24]
**171**	7.50×10^{6}	$HOCl + O_2^{\bullet-} \rightarrow Cl^\bullet + OH^- + O_2$	[24]
**172**	7.50×10^{6}	$HOCl + HO_2^\bullet \rightarrow Cl^\bullet + H_2O + O_2$	[24]
**173**	8.80×10^{9}	$OCl^- + HO^\bullet \rightarrow OCl^\bullet + OH^-$	[24]
**174**	2.00×10^{8}	$OCl^- + O_2^{\bullet-} + H_2O \rightarrow Cl^\bullet + 2OH^- + O_2$	[24]
**175**	2.50×10^{9}	$2ClO^\bullet \rightarrow Cl_2O_2$	[24]
**176**	2.50×10^{9}	$2ClO^\bullet + H_2O \rightarrow HOCl + H^+ + ClO_2^-$	[24]
**177**	2.50×10^{9}	$2ClO^\bullet + OH^- \rightarrow OCl^- + H^+ + ClO_2^-$	[24]
**178**	1.00×10^{9}	$HO^\bullet + ClO^\bullet \rightarrow ClO_2^- + H^+$	[24]
**179**	6.30×10^{9}	$HO^\bullet + ClO_2^- \rightarrow ClO_2^\bullet + H^+$	[24]
**180**	4.00×10^{9}	$HO^\bullet + ClO_2^\bullet \rightarrow ClO_3^- + H^+$	[24]
**181**	1.30×10^{8}	$Cl_2^\bullet + ClO_2^- \rightarrow ClO_2^\bullet + 2Cl^-$	[24]
**182**	9.40×10^{8}	$ClO^\bullet + ClO_2^- \rightarrow OCl^- + ClO_2^\bullet$	[24]
**183**	5.70×10^{5}	$CO_3^\bullet + OCl^- \rightarrow CO_3^- + ClO^\bullet$	[24]
**184**	1.00×10^{10}	$H^+ + ClO_2^- \rightarrow HClO_2$	[24]
**185**	1.90×10^{8}	$HClO_2 \rightarrow H^+ + ClO_2^-$	[24]
**186**	5.30×10^{5}	$2HOCl \rightarrow Cl_2O + H_2O$	[24]
**187**	3.50×10^{7}	$Cl_2O + H_2O \rightarrow H^+ + 2HOCl$	[24]

References

(1) Kundu, A.; Pousty, D.; Vadivel, V. K.; Mamane, H. Cu-Coated Graphitic Carbon Nitride (Cu/CN) with Ideal Photocatalytic and Antibacterial Properties. *Carbon Trends* **2023**, *13*, 100307. https://doi.org/10.1016/j.cartre.2023.100307.

(2) Pousty, D.; Betzalel, Y.; Mamane, H. Lighting Up MS2 : Exploring Fluence Rates for Virus Inactivation. *AWWA Water Science* **2025**, *7* (4), e70033. https://doi.org/10.1002/aws2.70033.

(3) Keen, O. S.; Love, N. G.; Linden, K. G. The Role of Effluent Nitrate in Trace Organic Chemical Oxidation during UV Disinfection. *Water Research* **2012**, *46* (16), 5224–5234. https://doi.org/10.1016/j.watres.2012.06.052.

(4) Cao, Z.; Yu, X.; Zheng, Y.; Aghdam, E.; Sun, B.; Song, M.; Wang, A.; Han, J.; Zhang, J. Micropollutant Abatement by the UV/Chloramine Process in Potable Water Reuse: A Review. *Journal of Hazardous Materials* **2022**, *424*, 127341. https://doi.org/10.1016/j.jhazmat.2021.127341.

(5) Lu, N.; Gao, N.-Y.; Deng, Y.; Li, Q.-S. Nitrite Formation during Low Pressure Ultraviolet Lamp Irradiation of Nitrate. *Water Science and Technology* **2009**, *60* (6), 1393–1400. https://doi.org/10.2166/wst.2009.475.

(6) Buxton, V. G.; Greenstock, C. L.; Helman, W. P.; Ross, A. B. Critical Review of Rate Constants for Reactions of Hydrated Electrons, Hydrogen Atoms and Hydroxyl Radicals ($\cdot OH/\cdot O^-$ in Aqueous Solution. *J. Phys. Chem. Ref. Data;(United States)* **1988**, *17* (2).

(7) Yin, R.; Dao, P. U.; Zhao, J.; Wang, K.; Lu, S.; Shang, C.; Ren, H. Reactive Nitrogen Species Generated from Far-UVC Photolysis of Nitrate Contribute to Pesticide Degradation and Nitrogenous Byproduct Formation. *Environ. Sci. Technol.* **2024**, *58* (46), 20676–20686. https://doi.org/10.1021/acs.est.4c05332.

(8) Scharko, N. K.; Berke, A. E.; Raff, J. D. Release of Nitrous Acid and Nitrogen Dioxide from Nitrate Photolysis in Acidic Aqueous Solutions. *Environ. Sci. Technol.* **2014**, *48* (20), 11991–12001. https://doi.org/10.1021/es503088x.

(9) Wang, Y.; Yin, R.; Tang, Z.; Liu, W.; He, C.; Xia, D. Reactive Nitrogen Species Mediated Inactivation of Pathogenic Microorganisms during UVA Photolysis of Nitrite at Surface Water Levels. *Environ. Sci. Technol.* **2022**, *56* (17), 12542–12552. https://doi.org/10.1021/acs.est.2c01136.

(10) Wang, P.; Bu, L.; Wu, Y.; Duan, X.; Dionysiou, D. D.; Zhou, S. Reactivity of Reactive Nitrogen Species and Degradation Kinetics of Micropollutants in the UV/Monochloramine Process. *ACS EST Water* **2022**, *2* (8), 1422–1430. https://doi.org/10.1021/acsestwater.2c00173.

(11) Goldstein, S.; Lind, J.; Merényi, G. Chemistry of Peroxynitrites as Compared to Peroxynitrates. *Chem. Rev.* **2005**, *105* (6), 2457–2470. https://doi.org/10.1021/cr0307087.

(12) Scholes, R. C. Emerging Investigator Series: Contributions of Reactive Nitrogen Species to Transformations of Organic Compounds in Water: A Critical Review. *Environ. Sci.: Processes Impacts* **2022**, *24* (6), 851–869. https://doi.org/10.1039/D2EM00102K.

(13) Vione, D.; Maurino, V.; Minero, C.; Pelizzetti, E. Phenol Photonitration upon UV Irradiation of Nitrite in Aqueous Solution I: Effects of Oxygen and 2-Propanol. *Chemosphere* **2001**, *45* (6–7), 893–902. https://doi.org/10.1016/S0045-6535(01)00035-2.

(14) Scholes, R. C.; Prasse, C.; Sedlak, D. L. The Role of Reactive Nitrogen Species in Sensitized Photolysis of Wastewater-Derived Trace Organic Contaminants. *Environ. Sci. Technol.* **2019**, *53* (11), 6483–6491. https://doi.org/10.1021/acs.est.9b01386.

(15) Kim, D.; Lee, J.; Ryu, J.; Kim, K.; Choi, W. Arsenite Oxidation Initiated by the UV Photolysis of Nitrite and Nitrate. *Environ. Sci. Technol.* **2014**, *48* (7), 4030–4037. https://doi.org/10.1021/es500001q.
(16) Li, J.; Cassol, G. S.; Zhao, J.; Sato, Y.; Jing, B.; Zhang, Y.; Shang, C.; Yang, X.; Ao, Z.; Chen, G.; Yin, R. Superfast Degradation of Micropollutants in Water by Reactive Species Generated from the Reaction between Chlorine Dioxide and Sulfite. *Water Research* **2022**, *222*, 118886. https://doi.org/10.1016/j.watres.2022.118886.
(17) Mack, J.; Bolton, J. R. Photochemistry of Nitrite and Nitrate in Aqueous Solution: A Review. *Journal of Photochemistry and Photobiology A: Chemistry* **1999**, *128* (1–3), 1–13. https://doi.org/10.1016/S1010-6030(99)00155-0.
(18) Yan, S.; Liu, Y.; Lian, L.; Li, R.; Ma, J.; Zhou, H.; Song, W. Photochemical Formation of Carbonate Radical and Its Reaction with Dissolved Organic Matters. *Water Research* **2019**, *161*, 288–296. https://doi.org/10.1016/j.watres.2019.06.002.
(19) Zhang, Z.; Chuang, Y.-H.; Huang, N.; Mitch, W. A. Predicting the Contribution of Chloramines to Contaminant Decay during Ultraviolet/Hydrogen Peroxide Advanced Oxidation Process Treatment for Potable Reuse. *Environ. Sci. Technol.* **2019**, *53* (8), 4416–4425. https://doi.org/10.1021/acs.est.8b06894.
(20) Haygarth, K. S.; Marin, T. W.; Janik, I.; Kanjana, K.; Stanisky, C. M.; Bartels, D. M. Carbonate Radical Formation in Radiolysis of Sodium Carbonate and Bicarbonate Solutions up to 250 °C and the Mechanism of Its Second Order Decay. *J. Phys. Chem. A* **2010**, *114* (5), 2142–2150. https://doi.org/10.1021/jp9105162.
(21) Sharpless, C. M.; Page, M. A.; Linden, K. G. Impact of Hydrogen Peroxide on Nitrite Formation during UV Disinfection. *Water Research* **2003**, *37* (19), 4730–4736. https://doi.org/10.1016/S0043-1354(03)00442-1.
(22) Smulik, R.; Dębski, D.; Zielonka, J.; Michałowski, B.; Adamus, J.; Marcinek, A.; Kalyanaraman, B.; Sikora, A. Nitroxyl (HNO) Reacts with Molecular Oxygen and Forms Peroxynitrite at Physiological pH. *Journal of Biological Chemistry* **2014**, *289* (51), 35570–35581. https://doi.org/10.1074/jbc.M114.597740.
(23) Gutknecht, J.; Bisson, M. A.; Tosteson, F. C. Diffusion of Carbon Dioxide through Lipid Bilayer Membranes: Effects of Carbonic Anhydrase, Bicarbonate, and Unstirred Layers. *The Journal of general physiology* **1977**, *69* (6), 779–794. https://doi.org/10.1085/jgp.69.6.779.
(24) Grebel, J. E.; Pignatello, J. J.; Mitch, W. A. Effect of Halide Ions and Carbonates on Organic Contaminant Degradation by Hydroxyl Radical-Based Advanced Oxidation Processes in Saline Waters. *Environ. Sci. Technol.* **2010**, *44* (17), 6822–6828. https://doi.org/10.1021/es1010225.
